# Psychotropic medications versus non-pharmacologic approaches for managing behavioural and psychological symptoms in Australian aged care residents with dementia: general practitioners’ and physicians’ perspectives

**DOI:** 10.1177/20451253251387908

**Published:** 2025-10-28

**Authors:** Hunduma D. Ayeno, Mustafa Atee, Gizat M. Kassie, Vijayaprakash Suppiah, Imaina Widagdo, Tuan A. Nguyen

**Affiliations:** Quality Use of Medicines and Pharmacy Research Centre, Clinical and Health Sciences, Corner of North Terrace and Frome Rd, University of South Australia, Adelaide, SA 5001, Australia; Department of Pharmacy, Ambo University, Ambo, Ethiopia; The Dementia Centre, HammondCare, Osborne Park, WA, Australia; Sydney Pharmacy School, Faculty of Medicine and Health, The University of Sydney, Sydney, NSW, Australia; Centre for Research in Aged Care, School of Nursing and Midwifery, Edith Cowan University, Joondalup, WA, Australia; Curtin Medical School, Faculty of Health Sciences, Curtin University, Bentley, WA, Australia; Quality Use of Medicines and Pharmacy Research Centre, Clinical and Health Sciences, University of South Australia, Adelaide, SA, Australia; South Australian Health and Medical Research Institute (SAHMRI), Women and Kids, Adelaide, SA, Australia; Australian Centre for Precision Health (ACPreH), Clinical and Health Sciences, University of South Australia, Adelaide, SA, Australia; Quality Use of Medicines and Pharmacy Research Centre, Clinical and Health Sciences, University of South Australia, Adelaide, SA, Australia; Quality Use of Medicines and Pharmacy Research Centre, Clinical and Health Sciences, University of South Australia, Adelaide, SA, Australia; National Ageing Research Institute, Social Gerontology Division, Melbourne, VIC, Australia; School of Health Sciences, Swinburne University of Technology, Melbourne, VIC, Australia

**Keywords:** BPSD, non-pharmacological interventions, physicians, psychotropics, residential aged care

## Abstract

**Background:**

Psychotropic medications are often inappropriately prescribed for behavioural and psychological symptoms of dementia (BPSD), posing significant risks such as falls, stroke and death. Although non-pharmacological interventions (NPIs) are the first-line treatment for BPSD, their use in practice remains limited.

**Objectives:**

This study explored general practitioners’ (GPs’) and physicians’ perspectives on using psychotropic medications compared to NPIs for managing BPSD in Australian residential aged care homes (RACHs).

**Design:**

Semi-structured online in-depth interviews were conducted with GPs and physicians managing BPSD in Australian RACHs.

**Methods:**

The interviews were audio-recorded, transcribed and analysed using inductive thematic analysis, with transcripts coded using NVivo 14 to generate themes.

**Results:**

Four GPs and eleven physicians were interviewed, and four major themes emerged: (1) knowledge of best practices of BPSD management, (2) awareness of current challenges in BPSD management, (3) non-involvement, blame shifting and rationalisation: perceived reasons for the current challenges and (4) suggested solutions. GPs and physicians were aware of best practices in managing BPSD, highlighting the importance of NPIs as more effective first-line strategies, with psychotropic medications reserved as a last resort. They also admitted that psychotropic over-prescription and inadequate NPI implementation persisted in BPSD management in Australian RACHs. Physician participants often distanced themselves from and blamed the GPs, staff and relatives of residents with dementia for the current problems. Systemic barriers, including insufficient resources, limited care continuity and organisational structures, were also reported to hinder psychotropic deprescribing. Implementing NPIs was deemed to be impeded by inadequate training and low confidence in their effectiveness. The participants suggested strengthening workforce capacity, incentivising NPIs and encouraging interdisciplinary collaboration.

**Conclusion:**

The results highlighted the gap between GPs’ and physicians’ knowledge of best practices and actual prescribing practices for BPSD in Australian RACHs. Improved workforce and support for NPIs could reduce reliance on psychotropics and align BPSD management with best practices.

## Background

Behavioural and psychological symptoms of dementia (BPSD) are subjective, diverse and often unpredictable, comprising one or more symptoms, including but not limited to, hallucinations, delusions, agitation, aggression, depression, anxiety, apathy, pacing, wandering, repetitive questioning, insomnia, anorexia or hyperphagia and various inappropriate behaviours.^1,2^ These symptoms are estimated to affect up to 90% of individuals with dementia at some point during the progression of their illness.^2^ Their impact is profound, with one study linking BPSD to a greater care burden, including caregiver depression and psychological distress.^3^ Furthermore, a systematic review found that specific symptoms, such as agitation/aggression, psychosis, depression and apathy, were associated with significant healthcare costs.^4^ To manage BPSD, guidelines recommend prioritising non-pharmacological interventions (NPIs), such as individualised music therapy, over psychotropic medications (e.g. antidepressants, antipsychotics),^5,6^ except as a last resort in severe cases when psychotropic medications are deemed necessary to mitigate the risk of harm to self or others (e.g. caregivers).^7^ Psychotropic medications are drugs that affect the mind, emotions and behaviour and include antipsychotics (e.g. risperidone), antidepressants (e.g. citalopram), anxiolytics/hypnotics (e.g. benzodiazepines), anticonvulsants (e.g. carbamazepine) and anti-dementia medications (e.g. donepezil).^8^ The three psychotropic medication classes most frequently associated with restrictive practice in residential aged care homes (RACHs) are antipsychotics, antidepressants and benzodiazepines,^9^ despite their limited efficacy for BPSD.^10–12^ For example, studies have shown that there was no significant difference in the effectiveness between antidepressants and placebo for treating depression in dementia.^12,13^ Similarly, a meta-analysis reported that there were no statistically and clinically significant differences between atypical antipsychotics and placebo in treating BPSD.^14^ Another meta-analysis indicated that there was no significant difference in efficacy between benzodiazepines and placebo.^15^

Despite the limited efficacy, psychotropic medications are over-prescribed and often used inappropriately in BPSD management. A 2015 observational study in Dutch RACHs reported that only 10% of psychotropic medication prescriptions were therapeutically appropriate for managing BPSD.^16^ Similarly, a meta-analysis reported that psychotropic polypharmacy was common in RACHs, with 33% and 13% of residents with dementia concurrently receiving two or three and three or more psychotropic medications, respectively.^17^ These psychotropic medications included antipsychotics, antidepressants, anxiolytics, sedative-hypnotics, anti-dementia medications and anticonvulsants.^17^ An interview study in the UK and a survey study in Dutch RACHs indicated that psychotropic medications were often overused in RACHs, particularly in residents with dementia.^18,19^ Two Australian studies indicated that these medications were frequently prescribed inappropriately for people with dementia.^20,21^ A retrospective cohort study conducted in Australia further demonstrated a substantial amount of antipsychotics, benzodiazepines and antidepressants being prescribed to RACH residents, including those with dementia.^22^

A report by the Australian Institute of Health and Welfare found that the prescription of antipsychotics, antidepressants and benzodiazepines increased by 6%–12% for the new RACH entrants within 6 months after entry, compared to the 6 months before entry.^23^ Notably, as general practitioners (GPs) are the main prescribers of psychotropic medications for BPSD, the proportion prescribed by them increased, while the prescriptions by specialists decreased during the same period.^23^ In Australia, antipsychotics, one class of psychotropic medications, were used as regular medications for an average of 6–24 months to manage BPSD, despite recommendations from pharmacists or doctors to review their use in 62% of aged care residents with dementia.^24,25^ This duration was significantly longer than the current recommendation of 3 months for reviewing, ceasing or tapering regular antipsychotics for BPSD.^26,27^ In addition, off-label use of antipsychotics other than risperidone, including olanzapine and quetiapine for BPSD, was also common.^24,28,29^ The rate of pro re nata (PRN; as needed) prescriptions for antipsychotics among residents with dementia ranges from 14.3% to 23.7%.^24,28^ This is despite the clinical guidelines’ recommendation against PRN use of antipsychotics and benzodiazepines for BPSD management in this population.^9^

However, the use of psychotropic medications is known to be associated with various adverse events and harms. Previous meta-analyses found that antipsychotics were associated with high rates of falls, delirium, mortality^30^ and cognitive decline.^31^ In addition, the initiation of antipsychotics after admission to RACHs was associated with a higher mortality risk in residents with dementia.^32^ A randomised clinical trial indicated that treating one thousand people experiencing BPSD with an atypical antipsychotic for 3 months would result in symptom improvement for 91–200 people. However, this treatment would also result in 10 additional deaths, 18 additional cerebrovascular events (e.g. stroke) and 58–94 people experiencing additional gait disturbances.^33^ Antidepressants were also linked to sleep disturbance, bleeding risk and hyponatremia^9^ and benzodiazepines were associated with sedation, confusion, fall-related fractures and cognitive worsening.^34^

Given these risks, efforts to reduce or discontinue psychotropic medications have been reported. Previous studies suggest that discontinuation of psychotropics has been successful in most RACH residents with major neurocognitive impairment without exacerbating BPSD.^35–37^ Person-centred music intervention in Australian rural RACH was reported to result in a decreased need for psychotropic medications.^38^ In the HALT study in Australia, training nurse champions on person-centred NPIs was part of an intervention where antipsychotics were deprescribed in 74.2% of residents, including 98% of residents with dementia.^36^ In addition, a cross-sectional survey in Australian RACHs showed that most family members were amenable to deprescribing if they received adequate support from GPs.^39^

Despite this evidence, deprescribing these medications remains a major challenge in residents with dementia.^40^ There are several reasons for the persistent use of psychotropic medications. A quantitative survey conducted in Australia on GPs regarding psychotropic prescribing for BPSD indicated that pressure from nurses (91%) was the primary influence on their prescribing, followed by pressure from residents’ families (59%).^41^ While several qualitative and quantitative studies have examined psychotropic prescribing,^19,41–43^ no qualitative studies have investigated GPs’ and physicians’ perspectives on the role of psychotropic medications compared to NPIs for managing BPSD in Australian RACHs. Thus, this study was conducted to address this knowledge gap.

## Method

### Study design

This study employed an explorative qualitative design using in-depth interviews of GPs and physicians involved in managing BPSD at Australian RACHs. The online interviews were conducted between October 2023 and August 2024. The Consolidated Criteria for Reporting Qualitative Research (COREQ) statement was employed to guide the reporting of results (Supplemental Appendix 1).^44^

### Study setting and recruitment

The study setting was RACHs in Australia. The expression of interest form for participation in this study was shared with Australian aged care providers and professional organisations of physicians. The flyer was also posted on LinkedIn and the research volunteers’ websites affiliated with the study. Purposive, convenience and snowball sampling techniques were employed to recruit study participants. The research team members (TN, MA, HA) played an active role in recruiting eligible GPs and physicians from across Australia.

### Inclusion and/or exclusion criteria

The interviewees, including GPs and physicians (e.g. geriatricians, old age psychiatrists) involved in managing BPSD at Australian RACHs, were eligible. No specific length of experience was needed to participate in the study.

### Data collection

Informed by the literature, a draft of an in-depth interview guide (Supplemental Appendix 2) containing semi-structured questions was prepared by the first author (HA).^43,45–47^ The draft was then sent to three field experts, who are members of the research team, including one industry advisor (second author), for face and content validation. These experts collectively had expertise in aged care, dementia, BPSD and clinical pharmacy. They reviewed the guide for clarity of the questions, relevance, logical flow, sensitivity and feasibility within the expected interview duration (with in 60 min). Feedback from the experts was incorporated into the guide, after which the first author (HA) piloted it with two independent researchers with a pharmacy background via mock interviews. The feedback received from these pilot testers further improved the interview guide before it was finalised. The guide consisted of two sections: (1) GPs’ and physicians’ thoughts and opinions on the role of psychotropic medications compared to NPIs for BPSD management, including when to use them, their effectiveness and factors influencing NPI implementation; and (2) GPs’ and physicians’ views on factors influencing psychotropic medication deprescribing and changes needed to optimise the use of both psychotropic medications and NPIs. The guide contained open-ended questions with certain probing when needed.

After obtaining consent from participants, the first author (HA) conducted and audio-recorded the online one-to-one interviews using Zoom or Microsoft Teams. The interviews continued until data saturation was achieved (i.e., no new information emerged with additional interviews). The data saturation point was reached with the 12th participant, as the final three participants did not provide additional information. Verbatim transcriptions were undertaken after each interview using the Microsoft Office Word transcribing function, accessed by the primary author through his affiliated University. The interview transcripts were verified against the original recordings by the first author.

For each interview, a one-page summary sheet was compiled after the first author thoroughly reviewed the transcript to understand the content. This preliminary analysis (one-page summary) was conducted by the first author in consultation with the last author for each interview to guide data collection in the subsequent interviews. The one-page summary and the transcript from each interview together formed one interview record for the final analysis.

### Data analysis

Thematic analysis, as outlined by Braun and Clarke^48,49^ was used to analyse the data and NVivo 14 software was employed for data coding. The analysis followed the six-phase framework developed by Braun and Clarke,^48,49^ which includes: (1) familiarisation with the data, (2) generation of initial codes, (3) searching for themes, (4) reviewing themes, (5) defining and naming themes and (6) producing the report. The analysis was also informed by literature suggesting that the use of a single coder is an acceptable and, indeed, common practice.^49^ This aligns with the nature of the study, as it is a PhD student project in which most work is carried out by the student, with supervisors providing guidance and supervision. In the first phase, the first author carefully read and re-read the transcripts to ensure familiarity with the data. In phase II, to ensure correct coding procedures were followed, the first author (HA) and the third author (GK, who has experience in qualitative research) independently coded one of the most information-rich transcripts as an exemplar. The discrepancies were resolved through consensus, which was used to develop a coding scheme. Using this coding scheme, the first author coded the remaining transcripts using NVivo 14 software and shared them with the research team (TN, MA, GK) for their review and feedback. In phase III, after incorporating feedback on coding from the research team, the first author identified themes and subthemes and shared them with the research team (TN, MA, GK, VS, IW) for further review and feedback. In phase IV, the first author revised the themes and subthemes after incorporating the comments received and shared them again with the team (TN, MA, GK, VS, IW) for additional input. In phase V, the first author drafted the names and descriptions of the themes and shared them with the research team (TN, MA, GK, VS, IW) for their review and feedback. In phase VI, the first author incorporated the comments and prepared the final draft of the manuscript, which was then circulated among the research team for their review and comments. This process continued until the manuscript was ready for submission.

### Study rigour and reflexivity

The interviewer, HA, is trained in credible qualitative data analysis and NVivo. No prior relationship existed between the interviewer and the interviewees. The interviewer had no known biases or assumptions that could have influenced the interviews. The interviewees were encouraged to speak openly and to ensure anonymity; each participant was assigned an alphanumeric code.

## Results

### Demographic characteristics

Of the 20 individuals who showed interest in participating in the interviews, 15 took part in the study, with each interview lasting between 24 and 60 min (average 38 min). Table 1 shows the demographic characteristics of the interview participants.

**Table 1.** Demographic characteristics of interview participants (*n* = 15).

Variables	Category	*n* (%)
Gender	Male	8 (53.3)
	Female	7 (46.7)
Specialty	GP	4 (26.7)
	Geriatrician	6 (40.0)
	Psychiatrist	4 (26.7)
	Palliative medicine specialist	1 (6.7)
Experience in the management of BPSD (years)	⩽5	2 (13.3
	6–10	2 (13.3)
	11–15	2 (13.3)
	>15	9 (60.0)
Location of RACHs^a^	Metropolitan	13 (86.7)
	Rural	5 (33.3)
State of practice location in Australia	SA	4 (26.7)
	NSW	5 (33.3)
	VIC	5 (33.3)
	TAS	1 (6.7)

a Three reported working in both metropolitan and rural settings.

GP, General practitioner; NSW, New South Wales; RACHs, Residential aged care homes; SA, South Australia; TAS, Tasmania; VIC, Victoria.

### Themes

Four themes were generated from the 15 interviews. These were: (1) knowledge of best practices in BPSD management, (2) awareness of current challenges in BPSD management, (3) non-involvement, blame-shifting and rationalisation: perceived reasons for challenges in BPSD management and (4) solutions suggested to optimise BPSD management.

### Knowledge of best practices of BPSD management

#### Knowledge regarding the importance of judicious assessment of BPSD triggers

Participants reported that careful and comprehensive evaluation of BPSD triggers should be conducted before tailoring interventions to individual needs. This evaluation could help address reversible causes of symptoms and determine whether psychotropic medications are necessary. One general practitioner **(P7)** stated: “*There should be a description of what of when, what behaviour would trigger the PRN, unfortunately, if you just write PRN and you don’t explain, then often the nurses will give it all the time*.” Similarly, a geriatrician **(P9)** highlighted the importance of investigating the underlying causes of behaviours, explaining: “*I want to try and figure out what the driver of the actual underlying causes for the behaviour. . . It would be important to liaise with the nursing staff to try and figure out what potentially is causing*.” A psychiatrist **(P4)** echoed this and emphasised the value of a multidisciplinary assessment in gathering accurate information, stating: “*Having access to an accurate and detailed multidisciplinary assessment. . . informed not only by observation of the person themselves but by talking to the various care staff*” (Supplemental Material 1).

#### Knowledge of best practices regarding the place of both psychotropic and non-pharmacological interventions

All interview participants who mentioned the role of psychotropic medications emphasised that these medications were to be used as a last resort and only as adjuncts, when necessary. In contrast, NPIs were considered the first line for the management of BPSD. A geriatrician **(P1)** stated: “*For me, it (psychotropic medication) is the last resort*,” and a GP **(P7)** added: “*Bringing in psychotropic medication when the non-pharmacological is not working*” (Supplemental Material 1). However, participants acknowledged the importance of psychotropics in specific situations, such as when a resident is very distressed, aggressive, agitated and experiencing severe psychosis, delusion, depression and anxiety (Supplemental Material 1). For example, a GP **(P7)** stated: “*Only if a situation becomes unsafe for the resident or for other residents, I would certainly be looking at psychotropic medication.*” In addition, a psychiatrist **(P8)** confirmed: “*Pharmacological should only be used if the situation is urgent or if the person was non-responsive to the non-pharmacological strategies.*”

As for the NPIs, one GP **(P15)** noted: “I think you tried to exhaust every non-pharmacological avenue first,” and a geriatrician **(P1)** stated: “All the very standard, non-pharmacological methods of addressing BPSD I would see first” (Supplemental Material 1).

#### Knowledge about the efficacy and safety of psychotropic and non-pharmacological interventions

##### Efficacy and safety of psychotropic medications compared to those of NPIs

Participants reported on the efficacy and safety of psychotropic medications in comparison to NPIs. From their experience, one GP **(P7)** stated: “*I do think that those medications [psychotropics] can calm people down. . . if there is an issue for their[residents’] safety and the safety of others*” and a psychiatrist **(P2)** added: “*So, they [psychotropic medications] definitely do have a role they do help I’ve seen them help I know that they make a difference*” (Supplemental Material 1). However, participants considered NPIs as a more effective and safer option than psychotropic medications for managing BPSD. For example, a geriatrician **(P3)** reported on the efficacy of NPIs stating: “*Some of the emerging data is that generally non-pharmacological has got more evidence of efficacy than that [antipsychotic].*” A psychiatrist **(P5)** confirmed: “*It [psychotropic medication] is less effective than non-pharmacological interventions. There’s not, actually a research base that medications are going to help with that [calling out].*” A palliative medicine specialist **(P6)** also stated: “*Net benefit is going to be far less than using non-pharmacological strategies*” (Supplemental Material 1).

Regarding the safety of psychotropic medications, participants reported that these medications could have adverse effects, including extrapyramidal side effects, confusion, agitation, hyponatremia, stroke and death. For example, one geriatrician **(P1)** admitted: “*We had to put him on quite a high dose. It (a high dose of risperidone) worked. He [the patient] settled, but he started to develop extrapyramidal symptoms and he was slowing down and clearly becoming Parkinsonian.*” *Making a comparison with NPIs*, a psychiatrist **(P5)** noted: “*A lot of the non-pharmacological interventions don’t really come with a lot of significant risks. It suggests that non-pharmacological approaches . . .are less likely to cause side effects*” (Supplemental Material 1).

#### Awareness of participants regarding the current recommendations of BPSD management guidelines

Participants were aware of clinical practice guidelines^9^ recommendation regarding a 12-week review period for psychotropic medications like risperidone. However, they also indicated that decisions about continuing or adjusting medications should be made based on the progress of the residents’ conditions. A geriatrician **(P3)** stated: “*You know the government here, requires you to continue, say risperidone. . . . 12 weeks before review, but in practice, people stay on drugs a lot longer.*” A General practitioner (**P7)** noted: “*The guidelines definitely say you should review every 12 weeks, but they don’t say everybody should have it only for 24 weeks*” (Supplemental Material 1).

#### Awareness of current challenges in BPSD management

##### Inadequate assessment of BPSD triggers

Participants highlighted that addressing underlying triggers, such as pain, was often overlooked in favour of increasing psychotropic medication(s), particularly in RACHs, where comprehensive assessments may be challenging. For example, a psychiatrist **(P2)** noted:

I often see cases where the GP is increasing the use of the psychotropic, so we’ll be increasing the quetiapine for instance. But hasn’t actually assessed pain in that person, but that (addressing triggers) doesn’t necessarily happen easily, particularly in residential aged care

##### Inadequate implementation of NPI

Participants emphasised the challenges of inadequate implementation of NPIs in RACHs. For example, a psychiatrist **(P2)** highlighted: “*Environmental factors, overcrowding, lack of access to quiet space and lack of access to outdoor space, those issues are not addressed adequately in residential aged care.*” A geriatrician **(P10)** also stated: “*It’s just that the staff would rather give a pill than do the hard work of putting in place a behavioural approach.*”

##### Overprescribing of psychotropic medications

Participants acknowledged the overprescribing practice of psychotropic medications in residents with dementia. For example, a geriatrician **(P10)** stated: “*Psychotropic medications are often overused and used as first-line rather than second-line agents*,” while another geriatrician **(P12)** confirmed this by saying:

You know, long-term use of SSRIs [selective serotonin reuptake inhibitors] among all the people, it’s very common for questionable indications . . . someone might be on more than one psychotropic. That’s . . . for their depression and that’s for their insomnia and that’s for their BPSD, whereas in fact, they all . . .roll into one

#### Non-involvement, blame-shifting and rationalisation: Perceived reasons for challenges in BPSD management

##### Distancing and blame-shifting regarding psychotropic prescribing

Physicians blame shifted the psychotropic medications overprescribing onto RACHs and their staff, residents and their families and the regulatory environment (Supplemental Material 2). A geriatrician **(P3)** reported: “*They (staff) feel more reassured when the person gets more medication because they perceive that these incidents are less likely to happen. . . . some of the government requirements make them(staff) fearful.*” Another geriatrician **(P1)** stated, “*. . . families are saying ‘if you don’t prescribe this medication the facility is going to send Mum away and not allow her to come back’*” and the same geriatrician (**P1**) reported, “*. . .staff saying, ‘well, we can’t keep this man because he’s hitting our staff or hitting other residents*’.” Another geriatrician **(P12)** noted: “*They[staff] just . . . glaze over and what they want you to do is get script pad out of your book . . . and write a drug.*”

##### Distancing and blame shifting regarding psychotropic deprescribing

There was a tendency among doctors to declare non-involvement and shift blame to other doctors. Psychiatrist (**P2)** said, “*I often hear about cases where the GP hasn’t reviewed somebody for several months.*” A Geriatrician **(P12)** echoed this blame shifting to other previous doctors of residents, stating, “*you’ll inherit somebody’s on several psychotropics from a hospital. He doesn’t give you any information.*” There was also blame shifting onto the RACHs and their staff, residents and their families. According to the participants, there was a reluctance to deprescribe psychotropic medications among the residents and their families as well as RACH staff (Supplemental Material 2). A geriatrician (**P1)** reported on the reluctance of residents, stating: “*So, barriers can be the patient themselves, who doesn’t want to stop it [psychotropic medication]*” and a GP (**P14)** highlighted families’ reluctance to psychotropic deprescribing, stating: “*The family . . . don’t want you to mess up with the medications too much . . . you just don’t rock the boat too much.*” Another geriatrician **(P13)** stated: “*I had in the past nursing staff at facilities reluctant to make this[deprescribing] trouble or reducing and occasionally GPs as well.*” (Please refer to Supplemental Material 2 for more quotes).

##### Distancing and blame-shifting regarding non-pharmacological intervention prescribing

There were non-involvement and blame shifting to RACH staff regarding the implementation of non-pharmacological interventions. Geriatrician **(P12)** stated, “*I to be honest, I don’t try to. I’ve given up trying to get major nondrug treatments. . . . first of all, . . . I’m not a nurse or enough to it.*” Another participant, a psychiatrist **(P2)**, echoed: “*They’re [doctors] just simply not trained in non-pharmacological interventions. So, it makes sense that . . .they’re not likely to go to those as a first line of prescription.*”

At the same time, participants also shared their perceptions of the underlying reasons for the challenges in managing BPSD, as outlined below.

##### Challenges of assessing BPSD triggers

A thorough assessment of BPSD triggers was challenging due to various factors. For example, a psychiatrist **(P2)** identified time constraints as a barrier, stating: “*. . .everything’s under time pressure and GPs often struggle to manage things. Their assessments are quick and hurried.*” Another psychiatrist **(P4**) highlighted the lack of knowledge, limited time and economic infeasibility as barriers to BPSD assessments among participants, admitting:

. . .we’re not taught how to do it [assessing BPSD triggers] . . . we don’t have the time to do it [assessing BPSD triggers] . . .we’re not an economical way of doing those assessments because medical time is very expensive compared to allied health and nursing time.

##### Reasons for psychotropic overprescribing

Various factors were reported to contribute to the overprescribing of psychotropic medications. These include difficulties in deciding the appropriate time to prescribe psychotropic medications, structural barriers (e.g. lack of infrastructure for NPIs, lack of centralised medical records for residents, financial issues) and feasibility/convenience of PRN options over NPIs (Supplemental Material 3). One geriatrician **(P1)** highlighted the absence of centralised records, stating: “*Because if people are using private scripts, there’s no real record other than the residential aged care facility saying no, we have a patient on this. Different GPs use the medications differently.*” Another geriatrician **(P12)** noted: “*. . . there’s no infrastructure to actually map that person’s behaviour . . . So, what are you gonna do? You’re just gonna keep running the scripts. . .we don’t subsidise the CBT [cognitive behavioural therapy].*”

#### Barriers to psychotropic deprescribing

##### Systemic factors: insufficient human resources, time and financial incentives

Lack of resources, including access to GPs’ and physicians’ review, lack of time among GPs and inadequate remuneration for GPs were mentioned as barriers to psychotropic deprescribing (Supplemental Material 4). A GP **(P14)** stated: “*In rural area . . . Doctor . . .is on call all the time. . .So I think maybe that’s when the deprescribing might be a bit difficult . . . they would choose to just leave it on.*” Meanwhile, a psychiatrist **(P2)** confirmed that: “*That’s [monitoring psychotropics] very difficult in residential aged care because GPs won’t. They’ve got a lot of people to see. GPs. . . haven’t got a lot of time.*” Another GP **(P11)** said: “*GPs. . . retreated from aged care because of the remuneration.*”

##### Intention to avoid the negative consequences of deprescribing

Participants expressed concerns about the recurrence of symptoms and the fear of negative consequences associated with reportable incidents under the current regulatory framework (Supplemental Material 4). A psychiatrist **(P4)** noted that: “*Fear on the part of the doctor that once they have started something, that if they stop it, the behaviour will occur.*” A geriatrician **(P10)** confirmed that “*. . . there’s always risks that you can get recurrence of previously managed symptoms.*” A geriatrician **(P3)** also highlighted the effect of the regulatory environment, stating: “*Some of the government’s requirements for reporting make staff quite fearful about weaning drugs.*”

The other barriers to deprescribing were the lack of guidance to psychotropic deprescribing, lack of flexible solid dosage forms to facilitate dose tapering, GPs’ reluctance to follow pharmacist recommendations due to prior negative experience, lack of knowledge and experience among GPs, physicians and care staff and lack of access to a continuum of care from specialists (please refer to the quotes for these barriers in Supplemental Material 4).

#### Barriers to prescribing non-pharmacological interventions

##### Lack of competence in NPIs among GPs, physicians and RACH staff

Lack of sufficient knowledge, education, training and experience on NPIs among GPs, physicians and RACH care staff were mentioned as barriers to implementing NPIs (Supplemental Material 5). For example, a psychiatrist **(P8)** stated: “*They [GPs] don’t have the knowledge and they don’t have the confidence, I guess, to do it [NPI]. And nurses aren’t very good at doing this either.*” Another psychiatrist **(P4)** confirmed that:

A lack of training and education about those (non-pharmacological) interventions during their [doctors’] undergraduate medical training and subsequent specialist training. . . .even their postgraduate specialty training doesn’t really equip them with their understanding of a) how to implement non-pharmacological measures and b) how to assess which non-pharmacological measures may or may not be appropriate.

##### A dominant biomedical model in BPSD management

The participants believed that there was an inherent bias among GPs and physicians towards psychotropic prescribing, influenced by their training and habitual practices. A psychiatrist **(P2)** stated: “*The doctors are trained in a medical model. They are prescribers and a lot of the time, the medications are the tools of their trade.*” A geriatrician **(P1)** reported scepticism about the evidence for NPIs efficacy as a barrier, saying: “*I think the evidence for the use of other things such as music therapy or exercise is not so well known.*” Another geriatrician **(P10)** confirmed that “*lack of trust in the intervention [NPI]*” was one barrier to NPI implementation (Supplemental Material 5).

##### Resource constraints

Lack of resources, such as insufficient trained staff, time constraints, lack of specialist dementia units and lack of funding were mentioned as barriers to prescribing NPIs (Supplemental Material 5). For example, a geriatrician **(P3)** reported: “*Prescriptions for non-pharmacological rely on more staffing than we have, so someone might need you know several hours of one-on-one time and in residential care that’s just not possible given the ratios.*” Another geriatrician (**P12)** highlighted the unsuitability of the current RACHs for BPSD management, saying: “*Really hard and you know the whole structural residential aged care is not designed to provide skilled dementia care.*”

Other barriers to NPI prescribing were a lack of access to in-person expert support and the nature and severity of resident behaviours (please refer to the quotes for these barriers in Supplemental Material 5).

#### Solutions suggested to optimise the management of BPSD

Participants suggested optimising the workforce of GPs, physicians and RACH staff as well as incentive strategies as potential solutions. One GP **(P7)** emphasised the need for well-equipped staff, saying: “*We need well-trained staff who can do the non-pharmacological management and who also understand dementia. And doctors, GPs and others the same who understand dementia and understand non-pharmacological management.*” A psychiatrist **(P2)** suggested one way to attract GPs to the RACHs, stating: “*Making sure that . . . there’s a sort of a business model that works for GPs to have enough time.*” Another psychiatrist **(P4)** explained the need to incorporate dementia courses into the undergraduate curriculum, stating:

I’ve highlighted the shortcoming of education with the sector where the sector might have at 50% staff turnover in 12 months, . . . but keep doing it [education for GPs and staff at RACH]. And unless you entrench the dementia-specific knowledge in peoples’ prevocational qualifications, it’s a doomed exercise. (Supplemental Material 6)

Other suggested strategies included increasing funding for education and staffing, optimising the RACH physical environment, implementing a person-centred approach, ensuring continuity of care, strengthening residential medication management review (RMMR), improving collaboration between GPs, physicians and other stakeholders, establishing partnerships between GPs, physicians, nurses and pharmacists and medication repurposing (please refer to the quotes for these strategies in Supplemental Material 6).

## Discussion

Although there were qualitative or quantitative studies focused on psychotropic prescribing,^19,41–43^ to the best of our knowledge, this is the first qualitative study to explore the views of GPs and physicians on the role of psychotropic medications in comparison to NPIs for managing BPSD in Australian RACHs. Four major themes emerged, namely (1) knowledge of best practices of BPSD management, (2) awareness of current challenges in BPSD management, (3) non-involvement, blame shifting and rationalisation: perceived reasons for the current challenges and (4) suggested solutions. GPs and physicians were aware of best practices in managing BPSD, highlighting the importance of NPIs as more effective first-line strategies, with psychotropic medications reserved as a last resort. They also admitted that psychotropic over-prescription and inadequate NPI implementation persisted in BPSD management in Australian RACHs. Physician participants often distanced themselves from and blamed the GPs, staff and relatives of residents with dementia for the current problems. Systemic barriers, including insufficient resources, limited care continuity and organisational structures, were also reported to hinder psychotropic deprescribing. Implementing NPIs was deemed to be impeded by inadequate training and low confidence in their effectiveness. The interviewees suggested strengthening workforce capacity, incentivising NPIs and encouraging interdisciplinary collaboration.

Regarding BPSD management best practices, GPs and physicians emphasised the importance of a multidisciplinary assessment of reversible triggers for BPSD as a crucial step in tailoring need-based interventions. This finding was consistent with a web-based survey study conducted by Cohen-Mansfield and Jensen, where physicians (more than half of whom were geriatricians) reported contacting at least one RACH staff to assess the resident’s situation.^50^ However, in our study, assessing BPSD triggers was reported to be time-consuming and not feasible for GPs and physicians without structural and organisational support. Participants reported not having enough time to spend in the RACH to assess BPSD triggers and noted that they were typically contacted only when a problem arose. Even if they had enough time for BPSD assessment, GPs and physicians felt they lacked the knowledge and skills to identify BPSD triggers and implement individualised NPIs.

Participants recognised NPIs as the first-line approach for managing BPSD, offering fewer or no side effects and greater benefits compared to psychotropic medications. They were aware that psychotropic medications should only be used as a last resort for severe BPSD, such as severe psychosis, aggression and agitation, particularly when there is a risk of harm to residents or staff and when NPIs have been adequately trialled and found ineffective. However, they also thought that the current strong, dominant biomedical model in dementia care and BPSD management with GPs’ and physicians’ trust in medications and distrust in the effectiveness of NPIs could hinder NPI implementation. In addition, a lack of competence among GPs, physicians and RACH staff, along with resource constraints such as limited numbers of trained staff, limited funding and time constraints, were also reported as barriers to NPI implementation. This was aligned with a study conducted by Wood-Mitchell et al.^42^ and Kerns et al,^43^ where lack of resources such as manpower, time constraints, skill mix and lack of evidence for NPI efficacy were identified as factors influencing NPI implementation. The GPs’ and physicians’ distrust in the effectiveness of some NPIs (e.g. music therapy, exercise) was consistent with a study conducted in Belgium, which revealed that GPs perceived NPIs, unspecified in the study, as insufficient treatment options for BPSD.^51^ The limited number of staff as a barrier in our study was also aligned with the findings of a quantitative survey study, where GPs reported that a lack of nursing staff at RACHs was the major barrier to their recommendation of NPIs for BPSD.^41^ The issue of funding constraints also emerged in a study conducted by Sawan et al.,^52^ where RACH managers complained about budget constraints in staff recruitment.

Participants acknowledged that psychotropic medications were not always inappropriate but emphasised that the problem was in their common misuse for managing BPSD. For instance, they observed that RACH staff often defaulted to using as-needed (PRN) psychotropics once prescribed, rather than implementing high-quality and evidence-based NPIs. Participants also recognised the issue of psychotropic overprescribing, attributing it to the lack of centralised records for residents with dementia, emotional pressure from families and staff to prescribe psychotropic medications, insufficient infrastructure for NPIs and financial challenges in subsidising them. Our finding related to psychotropic medication overprescribing was consistent with the findings from Donyai, where it was reported that there was a large group of residents on antipsychotics GPs and physicians were still less afraid to use them than before.^19^ The blame placed by GPs and physicians on families and staff for pressuring them to prescribe was consistent with another UK study, where an old age psychiatrist felt pressure from families, nurses and RACH staff.^42^ It is also aligned with the findings of a study conducted by Sawan et al.,^52^ where there were frequent requests for psychotropic medications as a solution for BPSD management due to work overload. In another study, GPs increased the dose of antipsychotics when requested to do so by family, residents or staff and most families opposed their cessation.^19^

In our study, participants acknowledged the role of psychotropic medication in managing BPSD. This finding was consistent with Donyai’s study, where a psychiatrist reported that antipsychotics made a significant difference and a RACH manager also noted that the benefits of antipsychotic medications typically appear after a few days of treatment, making residents more cooperative and relaxed.^19^ An online survey study indicated that half of the physicians surveyed agreed that antipsychotics had a positive calming effect on residents with dementia.^53^ Another survey study also revealed that physicians, nurses and family caregivers generally believed the potential benefits of antipsychotics outweighed the risk of side effects.^18^

Our participants also reported concerns about adverse effects associated with psychotropic medications. Despite these concerns, they admitted to continuing these medications for months to years, stopping only when behaviours of concern no longer existed. This aligned with the findings from a UK study where a psychiatrist noted that antipsychotics could be continued forever in residents with dementia.^19^ In that study, a community psychiatric nurse reported that most residents with dementia and their relatives preferred using psychotropic medications to manage BPSD, even when informed about their adverse effects.^19^ Another study found that primary care physicians trained in family medicine and internal medicine who cared for at least one resident in RACHs, continued prescribing antipsychotics despite the evidence of their adverse effects.^43^

Participants noted that BPSD guidelines had insufficient guidance on predicting and managing withdrawal symptoms, making it difficult to deprescribe psychotropic medications. In addition, systemic factors (insufficient human resources, lack of time and financial incentives), organisational factors (organisational culture), structural factors (lack of residents’ access to a continuum of care from specialists) and individual factors (intention to avoid the negative consequences of deprescribing, GPs’ and physicians’ reluctance to follow pharmacist recommendations due to prior negative experience and lack of knowledge and experience among GPs, physicians and care staff) were also identified as influencing the deprescribing of psychotropic medications. Our finding of barriers to psychotropic deprescribing, such as time constraints among GPs, the intention to avoid the negative consequences (e.g. concerns about symptom worsening), insufficient experience for deprescribing among GPs and physicians and a lack of implementation of pharmacists’ recommendations, is supported by a systematic review.^54^ Consistent with our findings, GPs feeling guilty about potentially worsening the residents’ condition, such as causing depression following the discontinuation of psychotropic medications, was also reported in another study by Van Leeuwen et al.^55^ Our study also found a lack of human resources as a barrier to deprescribing psychotropic medications, which was consistent with a study conducted in Denmark showing that high staff turnover, time constraints and lack of education among RACH staff hindered the deprescribing of antidepressants for BPSD.^56^ The intention to avoid the negative consequences as a barrier to psychotropic deprescribing was aligned with another study’s finding, which highlighted a dilemma between avoiding the oversedation of residents and protecting staff and other residents from harm resulting from dangerous behavioural symptoms.^57^ The lack of knowledge and experience among GPs, physicians and care staff was also a barrier, consistent with the findings of a study conducted in Belgium, where nurses with less education on antipsychotic deprescribing were a barrier to antipsychotic deprescribing.^51^

Participants suggested several strategies to improve NPI implementation and facilitate psychotropic deprescribing. They include providing incentives like salary increments and sustainable business models for GPs, optimising workforces (e.g. increasing the number of GPs, specialists and RACH staff), improving workforce knowledge and skills (e.g. educating and training RACH and medical staff on NPIs), government funding support for education and training, fostering collaboration between GPs, physicians and other professionals, as well as a partnership between GPs, physicians, nurses and pharmacists as potential strategies to optimise BPSD management.

## Strengths and limitations

The main strength of this study was the recruitment of GPs and physicians from four states of Australia with a mix of clinical specialties, genders and years of experience, with the majority having extensive experience in managing BPSD. However, most of the participants were working in metropolitan settings, which may result in limited generalisability to rural settings. Additionally, the interview guide was not reviewed by lived experience experts (i.e., residents with dementia or their carers. Another limitation is that, as the health care systems and prescribing regulations could vary significantly across the globe, the findings of this study should be interpreted cautiously in other countries. Given the increasing extent of nurse practitioners prescribing therapies for BPSD in Australian RACHs,^58^ future studies should include the voice of this stakeholder, adding to GPs’ and physicians’ perspectives to have a comprehensive picture of BPSD management practices in Australian RACHs.

## Conclusion

Study participants were aware of best practices in managing BPSD, highlighting the importance of NPIs, which were widely regarded as more effective, with psychotropic medications considered as a last-resort option reserved for emergencies. Despite this knowledge of best practices, participants reported that over-prescription of psychotropic medications remained common in BPSD management in RACHs in Australia. In addition, they advocated for prioritising NPIs over psychotropic medications and emphasised the importance of comprehensive and multidisciplinary assessments of BPSD to mitigate psychotropic prescribing. However, they also highlighted significant barriers to deprescribing and NPI implementation, suggesting that workforce optimisation and enhanced training are crucial for improving BPSD care.

## Supplemental Material

sj-docx-1-tpp-10.1177_20451253251387908 – Supplemental material for Psychotropic medications versus non-pharmacologic approaches for managing behavioural and psychological symptoms in Australian aged care residents with dementia: general practitioners’ and physicians’ perspectives Supplemental material, sj-docx-1-tpp-10.1177_20451253251387908 for Psychotropic medications versus non-pharmacologic approaches for managing behavioural and psychological symptoms in Australian aged care residents with dementia: general practitioners’ and physicians’ perspectives by Hunduma D. Ayeno, Mustafa Atee, Gizat M. Kassie, Vijayaprakash Suppiah, Imaina Widagdo and Tuan A. Nguyen in Therapeutic Advances in Psychopharmacology

sj-docx-2-tpp-10.1177_20451253251387908 – Supplemental material for Psychotropic medications versus non-pharmacologic approaches for managing behavioural and psychological symptoms in Australian aged care residents with dementia: general practitioners’ and physicians’ perspectives Supplemental material, sj-docx-2-tpp-10.1177_20451253251387908 for Psychotropic medications versus non-pharmacologic approaches for managing behavioural and psychological symptoms in Australian aged care residents with dementia: general practitioners’ and physicians’ perspectives by Hunduma D. Ayeno, Mustafa Atee, Gizat M. Kassie, Vijayaprakash Suppiah, Imaina Widagdo and Tuan A. Nguyen in Therapeutic Advances in Psychopharmacology

sj-docx-3-tpp-10.1177_20451253251387908 – Supplemental material for Psychotropic medications versus non-pharmacologic approaches for managing behavioural and psychological symptoms in Australian aged care residents with dementia: general practitioners’ and physicians’ perspectives Supplemental material, sj-docx-3-tpp-10.1177_20451253251387908 for Psychotropic medications versus non-pharmacologic approaches for managing behavioural and psychological symptoms in Australian aged care residents with dementia: general practitioners’ and physicians’ perspectives by Hunduma D. Ayeno, Mustafa Atee, Gizat M. Kassie, Vijayaprakash Suppiah, Imaina Widagdo and Tuan A. Nguyen in Therapeutic Advances in Psychopharmacology

sj-docx-4-tpp-10.1177_20451253251387908 – Supplemental material for Psychotropic medications versus non-pharmacologic approaches for managing behavioural and psychological symptoms in Australian aged care residents with dementia: general practitioners’ and physicians’ perspectives Supplemental material, sj-docx-4-tpp-10.1177_20451253251387908 for Psychotropic medications versus non-pharmacologic approaches for managing behavioural and psychological symptoms in Australian aged care residents with dementia: general practitioners’ and physicians’ perspectives by Hunduma D. Ayeno, Mustafa Atee, Gizat M. Kassie, Vijayaprakash Suppiah, Imaina Widagdo and Tuan A. Nguyen in Therapeutic Advances in Psychopharmacology

sj-docx-5-tpp-10.1177_20451253251387908 – Supplemental material for Psychotropic medications versus non-pharmacologic approaches for managing behavioural and psychological symptoms in Australian aged care residents with dementia: general practitioners’ and physicians’ perspectives Supplemental material, sj-docx-5-tpp-10.1177_20451253251387908 for Psychotropic medications versus non-pharmacologic approaches for managing behavioural and psychological symptoms in Australian aged care residents with dementia: general practitioners’ and physicians’ perspectives by Hunduma D. Ayeno, Mustafa Atee, Gizat M. Kassie, Vijayaprakash Suppiah, Imaina Widagdo and Tuan A. Nguyen in Therapeutic Advances in Psychopharmacology

sj-docx-6-tpp-10.1177_20451253251387908 – Supplemental material for Psychotropic medications versus non-pharmacologic approaches for managing behavioural and psychological symptoms in Australian aged care residents with dementia: general practitioners’ and physicians’ perspectives Supplemental material, sj-docx-6-tpp-10.1177_20451253251387908 for Psychotropic medications versus non-pharmacologic approaches for managing behavioural and psychological symptoms in Australian aged care residents with dementia: general practitioners’ and physicians’ perspectives by Hunduma D. Ayeno, Mustafa Atee, Gizat M. Kassie, Vijayaprakash Suppiah, Imaina Widagdo and Tuan A. Nguyen in Therapeutic Advances in Psychopharmacology

sj-docx-7-tpp-10.1177_20451253251387908 – Supplemental material for Psychotropic medications versus non-pharmacologic approaches for managing behavioural and psychological symptoms in Australian aged care residents with dementia: general practitioners’ and physicians’ perspectives Supplemental material, sj-docx-7-tpp-10.1177_20451253251387908 for Psychotropic medications versus non-pharmacologic approaches for managing behavioural and psychological symptoms in Australian aged care residents with dementia: general practitioners’ and physicians’ perspectives by Hunduma D. Ayeno, Mustafa Atee, Gizat M. Kassie, Vijayaprakash Suppiah, Imaina Widagdo and Tuan A. Nguyen in Therapeutic Advances in Psychopharmacology

sj-docx-8-tpp-10.1177_20451253251387908 – Supplemental material for Psychotropic medications versus non-pharmacologic approaches for managing behavioural and psychological symptoms in Australian aged care residents with dementia: general practitioners’ and physicians’ perspectives Supplemental material, sj-docx-8-tpp-10.1177_20451253251387908 for Psychotropic medications versus non-pharmacologic approaches for managing behavioural and psychological symptoms in Australian aged care residents with dementia: general practitioners’ and physicians’ perspectives by Hunduma D. Ayeno, Mustafa Atee, Gizat M. Kassie, Vijayaprakash Suppiah, Imaina Widagdo and Tuan A. Nguyen in Therapeutic Advances in Psychopharmacology

## Declarations

**Ethics approval and consent to participate:** Ethical approval was obtained from the University of South Australia Human Research Ethics Committee (Approval ID: 205134). All interview participants were given written information before providing informed consent to participate. Written informed consent was obtained from each participant before conducting the interviews.

**Consent for publication:** Not applicable, as there is no data identifying the participant. However, each participant was verbally informed at the end of their interview that the study would be published in a peer-reviewed journal.

**Competing interests:** Mustafa Atee is a Research and Practice Lead at The Dementia Centre; a research, education and consultancy arm of HammondCare, an independent Christian healthcare organisation which auspices the Dementia Support Australia (DSA) programs – the Dementia Behaviour Management Advisory Service (DBMAS) and the Severe Behaviour Response Teams (SBRT)’. These programs embrace and use multimodal, person-centred psychosocial and non-pharmacological interventions as part of their support strategies. He is one of the co-inventors of the original PainChek^®^ instrument (branded ePAT at the time), which was acquired and subsequently commercialised by PainChek Ltd. He is a shareholder of PainChek Ltd. Mustafa Atee previously held the position of a Senior Research Scientist (October 2018–May 2020) at PainChek Ltd. He co-authored a patent titled ‘A pain assessment method and system; PCT/AU2015/000501’ which was assigned to PainChek Ltd. and who have, to date, received granted patents in China, Japan, the European Union and the USA. The PainChek^®^ instrument was not mentioned nor cited in this manuscript. The remaining authors have no conflict of interest.

**Availability of data and materials:** Data supporting the findings of this study can be obtained from the corresponding author (HA) upon reasonable request.
