# Supplementary material for: Psychotropic medications versus non-pharmacologic approaches for managing behavioural and psychological symptoms in Australian aged care residents with dementia: general practitioners’ and physicians’ perspectives
Source: Ther Adv Psychopharmacol. 2025 Oct 28;15:20451253251387908. doi: 10.1177/20451253251387908 (PMC12575986; doi:10.1177/20451253251387908)
Supplement: sj-docx-2-tpp-10.1177_20451253251387908 – Supplemental material for Psychotropic medications versus non-pharmacologic approaches for managing behavioural and psychological symptoms in Australian aged care residents with dementia: general practitioners’ and physicians’ perspectives [file sj-docx-2-tpp-10.1177_20451253251387908.docx]

# Appendix 2: In-depth interview guide

1. **Participant selection**

Physician participants will be recruited from relevant physician associations, organisations, and professional networks through the distribution of a study participant expression of interest form, using the Qualtrics platform to relevant physician associations, organisations, and professional networks. Interested physicians will be expected to respond to the form by providing their names and email addresses for future communications for scheduling the actual in-depth interview.

We are aiming for about 20 physician participants, and the study participant recruitment will continue until it reaches this target number. All physician respondents will be contacted and provided with both the participant information sheet and consent form to sign and specify a suitable time for an in-depth interview. Only those who will provide the consent will be scheduled for an interview. At the end of each interview, we will utilise the snowball sampling technique by asking the interviewees to introduce their physician colleagues to the study to help reach the sample size.

1. **In-depth interview email confirmation letter**

Dear ________________,

Thank you for your willingness to participate in this in-depth interview. We would like to hear about your opinion and experience about psychotropic prescribing practice for the management of behaviours and psychological symptoms of dementia (BPSD) in Australian aged care homes. The in-depth interview will take about 45 minutes to a maximum of 1 hour to complete. Your responses to the questions will be kept anonymous. We will provide you with a 60 AUD Coles gift card in appreciation of your time at the end of the interview. We have attached the participant information sheet and consent form to this email for your review and signature. We kindly ask you to carefully read and sign the consent form either manually or electronically and then attach it in your reply to this email. Alternatively, you can reply to this email, confirming your agreement to participate in the interview by simply saying ‘yes’. Please let us know a convenient date and time for an interview so that we can schedule it using Microsoft Teams or Zoom and provide you with the corresponding link.

DATE __________________

TIME ___________________

If you need further clarification about the in-depth interview or will not be able to attend for any reason, please reply to this email with your inquiry or call 045xxxxx49. Otherwise, we look forward to your suitable time and date.

Sincerely,

Hunduma Ayeno, PhD Fellow

University of South Australia

1. **Welcome speech.**

Hello. Good afternoon. Nice to meet you and thank you very much for your time and for agreeing to participate in our interview today. I truly value your time and expertise. This interview should not be longer than 45 min to 1 hour. However, we may finish earlier.

Thank you for signing the consent form. Do you have any questions regarding the participant information sheet or consent form? The purpose of this interview is to get a better understanding of your opinions and thoughts on the place of psychotropic medication as compared to non-pharmacological interventions in the management of behaviours and psychological symptoms of dementia (BPSD) in residential aged care homes. This interview will be audio-recorded and transcribed for analysis. May I start an audio recording? I have formally started audio recording. Thank you once again for signing the consent form and giving me permission to audio record this interview. The audio recording will be transcribed for analysis and there will be no names attached to the transcript, so your words will remain anonymous. I have a series of questions and there are no right or wrong answers here, just your experience, thoughts, and insights. Please try to voice everything. The interview has three sections. Starting with the first section, some demographic information. The second section is about the role of psychotropic medicines in the management of BPSD at residential aged care homes. Lastly, the third section is about factors influencing the de-prescribing of psychotropic medicines for BPSD. Do you have any questions before I proceed to the interview questions? Thank you.

1. **In-depth interview questions**

**Section 1: Demographic information**

1. What is your specialty? **(Prompt:** General Practitioner, Geriatrician, Psychiatrist, Neurologist, …)
2. What State are you from?
3. What is/are the location(s) of the current residential aged care home where you have worked? (**Prompt:** Metropolitan, Rural, Remote)
4. What are your years of experience with BPSD/Dementia management in residential aged care home(s)?

**Section 2: This section is intended to explore your opinions and thoughts on** **the role of psychotropic medicines in the management of BPSD.**

1. What is the role of psychotropic medications in the management of BPSD in a RACH?

Probing:

- What is your perception of the effectiveness of psychotropic medications as compared to non-pharmacological interventions for managing BPSD?
- Under what circumstances psychotropic might be prioritized over non-pharmacological intervention for BPSD?
- What factors do you think enable or prevent physicians from prescribing non-pharmacological interventions to replace medications for BPSD management?
- In what circumstances are psychotropic medicines prescribed on a PRN (as needed) basis for BPSD? Could you provide some examples or scenarios where you believe it would be inappropriate to use this prescribing approach?
- In what circumstances would it be necessary to prescribe two or more psychotropic medicines simultaneously for BPSD?

**Section 3: This section is intended to explore your opinions and thoughts on the barriers to and enablers of psychotropic medicine de-prescribing in residents with BPSD.**

1. How long should psychotropic medications be prescribed?
2. In what circumstances do you think it would be better to de-prescribe a psychotropic medicine for BPSD?

Probing:

- Please share an example of a recent attempt you made to de-prescribe any psychotropic medicine for BPSD?
- Why did you decide to de-prescribe the psychotropic medicine?
- What was the outcome of your de-prescribing?

1. What do you think are the enablers of de-prescribing psychotropic medicine for BPSD?

Probing:

- Please give some common enablers as prompts if necessary.

1. What circumstances do you believe can lead to the prescribing of psychotropic medications for BPSD requiring duration longer than what is recommended by the current guidelines?

Probing:

- Please give some common barriers to psychotropic de-prescribing as prompts if necessary.

1. What is the role of the Residential Medication Management Review (RMMR) in psychotropic de-prescribing for BPSD at RACH?

- What needs to change to ensure appropriate use of psychotropic medications, and non-pharmacological interventions for BPSD management in RACH?

**Closing**

What else you would like to say about prescribing and de-prescribing of psychotropic medicines in management of BPSD in RACHs?

I will analyse the information provided by you and others, and I plan to publish it in a peer-reviewed journal.

If you are interested, I would be glad to share a copy with you for review once it is published.

Thank you very much for sharing your expertise on this topic.

Please feel free to introduce your physician colleagues for the next interviews.

Goodbye.
