# Supplementary material for: Psychotropic medications versus non-pharmacologic approaches for managing behavioural and psychological symptoms in Australian aged care residents with dementia: general practitioners’ and physicians’ perspectives
Source: Ther Adv Psychopharmacol. 2025 Oct 28;15:20451253251387908. doi: 10.1177/20451253251387908 (PMC12575986; doi:10.1177/20451253251387908)
Supplement: sj-docx-3-tpp-10.1177_20451253251387908 – Supplemental material for Psychotropic medications versus non-pharmacologic approaches for managing behavioural and psychological symptoms in Australian aged care residents with dementia: general practitioners’ and physicians’ perspectives [file sj-docx-3-tpp-10.1177_20451253251387908.docx]

**Supplementary material 1: Knowledge of best practices in BPSD management**

Supporting Information1**:** Knowledge of best practices in BPSD management

| Knowledge about the importance of judicious assessment of BPSD triggers |
| --- |
| *I want to try and figure out what the driver of the actual underlying causes for the behaviour… if they have a non-healing ulcer…causing pain then actually treat pain appropriately…would potentially deescalate behaviours...We can see anxiety and depression in people that then come out as behaviours as well... look for an underlying cause…It would be important to liaise with the nursing staff to try and figure out what potentially is causing. (****P9, Geriatrician)*** *…looking at triggers looking at… more of a life course approach. (****P10, Geriatrician)*** *So, we try to make sure we're excluding physical problems… pain, dental infection. (****P11, GP)*** *There should be a description of what of when, what behaviour would trigger the PRN,* *unfortunately, if you just write PRN and you don't explain, then often the nurses will give it all the time.* ***(P7, GP)*** |
| *Having access to an accurate and detailed multidisciplinary assessment, I have dementia consultants conduct that[assessment] before they bring that case to me. Their view is informed not only by observation of the person themselves, but by talking to the various care staff. (****P4, Psychiatrist)*** |
| *Knowledge of best practice regarding the use of both psychotropic and non-pharmacological interventions.* |
| **Psychotropic medications as a last resort**  *For me, it (psychotropic medication) is the last resort.* ***(P1, Geriatrician)*** *It[psychotropic] shouldn't be the first line, P****5, Psychiatrist.*** *Bringing in psychotropic medication when the non-pharmacological is not working. (****P7, GP)*** *They (psychotropic medications) should be a second-line choice …should never be the first line of treatment unless it's for a very targeted symptom. (****P2, Psychiatrist)****. Drug treatment has to be the last line. (****P3, Geriatrician)*** *So, it [psychotropic medication] shouldn't be the first line, it can be there as an adjunct to other measures. (****P9, Geriatrician)*** *But I don't recommend them (psychotropics) routinely. Yeah, I would be recommended as a second or third option.* ***(P13, Geriatrician)*** |
| NPIs as first-line  *All the very standard, non-pharmacological methods of addressing BPSD I would see first. (****P1, Geriatrician)*** *They [NPIs] should be used as the first line of intervention for this [BPSD] particular problem. (****P2, Psychiatrist)*** *Except in the setting of severe psychosis, I think non-pharmacological got to be the first line. (****P3, Geriatrician)*** *Yeah, you always have to go for the non-pharmacological interventions first. … You'd want to try behavioural interventions first. (****P6, Palliative medicine specialist)*** *First I'd start non-pharmacological. (****P7, GP)*** *Then we tried non-pharmacological management and then we tried pharmacological management. (****P8, Psychiatrist)*** *I think the overall majority would probably benefit the first line from non-pharmacological. … Yes, the non-pharmacological measures will be first every time. (****P9, Geriatrician)*** *I think we should always be trialling the non-pharmaceutical approaches first. (****P10, Geriatrician)*** *You do the non-pharmacological first… before utilising any psychotropics. (****P14, GP)*** *A big, bigger practice try behaviour interventions as best you can and support before using pharmacological therapy. (****P11, GP)*** *I think you tried to exhaust every non-pharmacological avenue first…you try everything first you try non-pharmacological interventions.* ***(P15, GP)*** |
| *Knowledge about the efficacy and safety of psychotropic and non-pharmacological interventions* |
| Efficacy and safety of psychotropic medications |
| Efficacy  *If you look at … particularly of, say risperidone had a modest effect. (****P1, Geriatrician)*** *Efficacy say for antipsychotics might be something like a 10% reduction in aggression. (****P3, Geriatrician)*** *So, I don't think they're[psychotropics] as effective as the non-pharma stuff. (****P4, Psychiatrist)*** *It [psychotropic medication] is less effective than non-pharmacological interventions. There's not, actually a research base that medications are going to help with that [calling out]. (****P5, Psychiatrist)*** *Net benefit is going to be far less than using non-pharmacological strategies. (****P6, Palliative medicine specialist).*** |
| *I do think that those medications can calm people down… if there is an issue for their safety and for the safety of others.* ***(P7, GP)*** *So, they [psychotropic medications] definitely do have a role they do help I've seen them help I know that they make a difference. (****P2, Psychiatrist)*** *A large-scale observational study of people in aged care facility for managing psychological behavioural problems and when they did a very careful and large study and controlled by collecting data on all sorts of potential variables and confounders that might influence the outcomes and I think they used the outcome of being admitted to hospital as the outcome. Which it may be a slightly different outcome to what we would talk about normally, but they can. The study came to the conclusion that small doses of pharmacological agents often used in combination were more effective at keeping the patient cohort out of hospital. (****P11, GP)*** *Psychotropics because they have their place and they can keep patients and their families and people around them safe. It's very well, especially aged care facility.* ***(P15, GP)*** |
| Safety  *We had to put him on quite a high dose. It (a high dose of risperidone) worked. He (the patient) settled, but he started to develop extrapyramidal symptoms and he was slowing down and clearly becoming Parkinsonian. …* *it (antidepressant) is unfortunately lowering his sodium. (****P1, Geriatrician)*** *… very important, negative side effects from psychotropics like increased risk of stroke and death. (****P7, GP)*** *Another common one I see is clients that are on olanzapine, … for BPSD, particularly for agitation or aggression ... was on quite high doses of olanzapine … making them more confused. (****P5, Psychiatrist)*** *I think some of the hardest situations I see is where psychotropics have been used in Lewy body dementia with terrible effects, shocking adverse effects and I've seen it once or twice where it's been irreversible. (****P6, Palliative medicine specialist)*** |
| Drug-drug interaction  *A client that had a Parkinson's disease and then developed psychotic symptoms and the client was on medication for their Parkinson's disease but were also on antipsychotic medication and essentially the two medications were sort of fighting one another because one was replacing the client’s dopamine and then the other one was working as a dopamine blocker. (****P5, Psychiatrist)*** |
| Efficacy and safety of NPIs |
| Efficacy  *The very large body of data is that non-pharmacological strategies …are actually more effective. (****P2, Psychiatrist)*** *Some of the emerging data is that generally non-pharmacological has got more evidence of efficacy than that [antipsychotic]. (****P3, Geriatrician)*** *A paper looking at the effectiveness of non-pharmacological interventions a couple of years … showed reduction in [two-thirds of the cases].* ***(P4, Psychiatrist)*** *It suggests that non-pharmacological approaches have got more effectiveness. I'd say it [psychotropic medication] is less effective than non-pharmacological interventions. If manage the calling out with non-pharmacological interventions, you can potentially get a response and a benefit. (****P5, Psychiatrist)*** *The evidence is pretty clear that non-pharmacological is better than pharmacological. (****P8, Psychiatrist)*** *There's absolutely no doubt you can reduce the need for psychotropics by the appropriate use of you know, understanding the person's triggers and needs. You know there's good evidence that CBT can give you as just as an effective treatment for insomnia. (****P12, Geriatrician)***. |
| Safety  *Using non-pharmacological strategies there are very few harms. (****P6, Palliative medicine specialist)*** *A lot of the non-pharmacological interventions don't really come with a lot of significant risks. It suggests that non-pharmacological approaches …are less likely to cause side effects. (****P5, Psychiatrist)*** *“Using non-pharmacological strategies there's very few harms. (****P6, Palliative medicine specialist)*** *A lot of the non-pharmacological interventions don't really come with a lot of significant risks. It suggests that non-pharmacological approaches …are less likely to cause side effects. (****P5, Psychiatrist)*** |
| *Knowledge of the current BPSD guideline recommendation.* |
| *You know the government here, it requires to continue, say risperidone. … 12 weeks before review, but in practice, people stay on drugs a lot longer. (****P3, Geriatrician)*** *I think the guidelines are based on experience, not on trials… every person is an N of 1 trial.* *I've had people who've had to stay on antipsychotics for two years, but then eventually they come off them (****P1, Geriatrician)*** *so what we do in our facility, which is according to the guidelines is, we give them a trial of 12 weeks and then we review them. And so, we monitor the behaviour the outcomes of the medication. … but she still gets agitated sometimes. Let's try it for another 12 weeks. And because we know that with dementia the behaviour gets worse and then it slows down. Every person is different…I often use it … three or four lots of 12 weeks.* ***(P7, GP)*** *Would the guidelines do actually say 12 weeks? And then review…they don’t. The guidelines definitely say you should review every 12 weeks, but they don't say everybody should have it only for 24 weeks. Only for 36 weeks…OK, that's my understanding of the guidelines anyway. … And it does not put any deadline to stop that medication… The deadline would be if the behaviour diminishes and there's no behaviour anymore.* (**P7, GP)** *Your expectation of time like six months or so, whatever we decide. And if it is still needed then it's still needed.* ***(P9, Geriatrician)*** *If you've got someone who's got severe behaviour that needs multiple agents gets settled into a nursing home, the time you might consider it might be … between three and six months after admission, maybe six months after admission, you might try to gradually reduce the medications.****(P12, Geriatrician)*** *I understand that every six months or three months I've got to sign off on a chemical restraint form, … and you know sign that they need to continue the medication it is easy to start something. And not stop it. (****P13, Geriatrician)*** |

When to consider the prescribing of psychotropic medication for BPSD.

| **Conditions** | **Illustrations** |
| --- | --- |
| Emergency or urgency response to unsafe or distressing situations | *When … and there's some urgency to get things under control.* ***[P4, Psychiatrist]*** *… if people are starting to be at risk of harming themselves or others.* ***[P6, Palliative medicine physician]*** *… other residents or other staff members were at high risk.* ***[P1, Geriatrician]*** *…Only if a situation becomes unsafe for the resident or for other residents, I would certainly be looking at psychotropic medication.* ***[P7, GP]*** *… pharmacological should only be used if the situation is urgent or if the person nonresponsive to the non-pharmacological strategies.****[P8, Psychiatrist]*** *… if somebody is a danger to themselves or others.* ***[P8, Psychiatrist]*** *… for the safety of the individual, for the safety of the other residents and the staff.* ***[P9, Geriatrician]*** *… an emergency situation.****[P10, Geriatrician]*** *… when the person is at risk to themselves and also the residents and staff.* ***[P9, Geriatrician]*** *… when somebody is a significant risk to others.* ***[P13, Geriatrician]*** |
| *Very distressed resident* | *… someone who's very distressed.* ***[P3, Geriatrician]*** *… resident is very distressed by their symptoms.* ***[P1, Geriatrician]*** *… they've become much more disturbed… outbreaks of bad behaviour.* ***[P7, GP]*** *… in acute severe distress to the resident.* ***[ P10, Geriatrician]*** *… very distressed and agitated, scared.* ***[P14, GP]*** |
| Active psychotic symptoms | *… they've got very severe psychotic symptoms.* ***[P3, Geriatrician]*** *… severely distressed by their delusions or their hallucinations… person clearly was hallucinating. Or had delusions.* ***[P1, Geriatrician]*** *… management of psychotic symptoms.* ***[P4, Psychiatrist]*** *… reduce all of their persecutory delusions, and hallucinations.* ***[P15, GP]*** |
| Aggression and agitation | *… in aggression and agitation that pose an immediate risk.* ***[P4, Psychiatrist]*** *… the person is very agitated.* ***[P1, Geriatrician]*** *… Aggression is the other area.* ***[P1, Geriatrician]*** *… intermittent agitation.* ***[P8, Psychiatrist]*** *… escitalopram or citalopram, for the treatment of agitation… severe physical aggression, severe agitation… use an antipsychotic drug.* ***[P12, Geriatrician]*** *… an individual is physically aggressive…pharmacological options…more effective.* ***[P15, GP]*** |
| When NPI is not working | *… behaviour doesn't completely go with non-pharmacological interventions… add …a low dose perhaps of an antipsychotic.* ***[P1, Geriatrician]*** *… about bringing in psychotropic medication. When the non-pharmacological is not working.* ***[P7, GP]*** *… I'd start non-pharmacological … if that was not controlling… I would move on to psychotropic medications.* ***[P7, GP]*** *… prescribing it (NPI) to … depression and … non-responsive … trying … antidepressant.* ***[P8, Psychiatrist]*** *… when these have failed non-pharmacological measures… that would be the role of the medications.* ***[P9, Geriatrician]*** *… the role is when non-pharmacological strategies have failed.* ***[P13, Geriatrician]*** *… not able to you know control by non-pharmacological… that's when you would consider using pharmacological.* ***[P14, GP]*** *… but when the patient's physical risk. Is refractory to non-pharmacological measures... Then I would say ... oral is effective.* ***[P15, GP]*** |
| Prevention of anxiety and agitation | *… If a client has a history of anxiety prior to developing their dementia.* ***[P5, Psychiatrist]*** *… when someone's on the brink of being sent into hospital from a nursing home.* ***[P3, Geriatrician] …*** *if the person was going to be taken out, perhaps of the facility.* ***[P1, Geriatrician]*** *… I have prescribed them (PRN)…if am trying to stop something but people are really anxious about this stopping something… BPSD symptoms are preventing somebody from receiving care interventions.* ***[P13, Geriatrician]*** *… there is a history of that [distressing event] happening from time to time… we would usually leave the PRN medications on.* ***[P14, GP]*** |
| Prevent pain-related agitation | *…verbal or physical aggression in the context of personal care, perhaps because they're, you know, have a significant amount of pain.* ***[P5, Psychiatrist]*** |
| ***Indications for*** *more than one psychotropic medication* | |
| Add-on therapy during dose tapering of the primary drug | *…we got him down quite a long way to .25 milligrams of risperidone twice daily… which was. enough to take the edge off his delusions, but he was still quite agitated, so we actually added citalopram … that actually settled his agitation.* ***[P1, Geriatrician]*** |
| Uncontrolled severe depression | *Another one would be with the antidepressants, where after a year. I'd say venlafaxine because someone's quite severely depressed and they may have been on Mirtazapine as well… You know, that's called California rocket fuel the combination of venlafaxine and mirtazapine. [****P1, Geriatrician]*** |
| Bridging therapy for symptom control | *… someone might be on an antidepressant… then getting benzos or benzodiazepines temporarily while you put up the antidepressants, so you maintain their symptoms.* ***[P3, Geriatrician]*** |
| Anxiety or depression symptoms plus pain | *… I've alluded to I would consider pain medication the psychotropic medication, so there may be an example where you're prescribing an antidepressant because there are depressive symptoms or anxiety symptoms and you're also prescribing pain management.* ***[P5, Psychiatrist]*** |
| Preexisting depression plus BPSD | *… Quite a reasonable one perhaps … might be somebody who's prescribed an antidepressant for depression, as you know, been on that for years, …. But if they then develop BPSD, they might be prescribed in antipsychotic or benzodiazepine to help manage that [BPSD].* ***[P4, Psychiatrist]*** |
| Psychotic symptoms or aggression plus depression or pain | *…Occasionally there might be … a degree of psychotic symptoms or significant aggression and … comorbid depression or pain…in that scenario, you potentially could have an antidepressant being prescribed with an antipsychotic or some pain medication, alongside an antipsychotic.* ***[P5, Psychiatrist]*** *… I have prescribed 2 or more psychotropic medications where a person is severely depressed and so, you would prescribe an antidepressant for the depression but then you realise it's a psychotic depression. And usually, I would not be the one doing. I would take advice from a psychiatrist colleague. But often they will prescribe an antipsychotic along with the antidepressant and that certainly I have seen it being very effective.* ***[P1, Geriatrician]*** *… I don't think that's a bad thing. I think it's better to have two different medications at low doses… Like the lady who was crying, I put on Citalopram, which is also an antidepressant, but she was also on an antipsychotic because she was agitated.* ***[P7, GP]*** *… a psychotic depression or suicidal depression, then an antidepressant alone will not work and they usually need an antipsychotic as well.* ***[P8, Psychiatrist]*** … *You may use a more sedating antidepressant. But they may also have some…hallucinations for the usual thoughts, … in which case you may also want to be using an antipsychotic.* ***[P10, Geriatrician]*** *… you know the background of… depression or…psychotic depression… so that's where probably…we could prescribe both.* ***[P14, GP]*** |
| Dementia and depression | *…So, when somebody's like a cholinesterase inhibitor or memantine. And seen and gets prescribed … an antidepressant, which again is entirely appropriate. It's not chemical restraint, but they still get captured in that group of people who are prescribed two or more psychotropic agents.* ***[P4, Psychiatrist]*** |
| Mood disorder plus depression or psychosis | *…people who are on mood stabilisers because they're impulsive, but they could be having another symptom like depression or psychosis… The patient’s psychosis with this. And their depression with this other drug.* ***[P3, Geriatrician]*** *… certain psychiatric conditions, like bipolar disorder will need a mood stabiliser as well as an antidepressant or something to calm down. If they're in a manic phase.****[P8, Psychiatrist]*** |
| Lewy body dementia and psychotic symptoms. | *…if someone is on Donepezil, they're getting psychotic symptoms, … and they've got dementia with Lewy bodies, you might give them a cholinesterase inhibitor but also a low dose of antipsychotic.* ***[P3, Geriatrician]*** |
| Sleep disturbances plus agitation. | *…. you have you have somebody who's on a SSRI already or on a benzodiazepine for nighttime use and then you're dealing with, you're presented with a person who is very agitated during the day …And you've tried other things … to distract them, … you're then starting a low dose of a second-generation antipsychotic risperidone or something like that as a trial.* ***[P11, GP]*** |
| Tier 6 and 7 in the Brodaty triangle | *In the Brodaty triangle, certainly, T6 and T7 will need to be on multiple psychotropics. See, once you've crossed that rubicon of where you've had either interpatient or you had repeated staff assaults, or any patients also had severe behavioural problems, you will eventually end up on multiple agents.* ***[P12, Geriatrician]*** *… when somebody is particularly dangerous…*  *two or more psychotropic medications, particularly for … punching people, including other people and pushing them over and being with physical stress… People died.* ***[P13, Geriatrician]*** |
