# Supplementary material for: Psychotropic medications versus non-pharmacologic approaches for managing behavioural and psychological symptoms in Australian aged care residents with dementia: general practitioners’ and physicians’ perspectives
Source: Ther Adv Psychopharmacol. 2025 Oct 28;15:20451253251387908. doi: 10.1177/20451253251387908 (PMC12575986; doi:10.1177/20451253251387908)
Supplement: sj-docx-4-tpp-10.1177_20451253251387908 – Supplemental material for Psychotropic medications versus non-pharmacologic approaches for managing behavioural and psychological symptoms in Australian aged care residents with dementia: general practitioners’ and physicians’ perspectives [file sj-docx-4-tpp-10.1177_20451253251387908.docx]

| **Supplementary material 2: Non-involvement and blame shifting** |
| --- |
| Non-involvement and blame shifting regarding psychotropic prescribing |
| Blame shifted onto residents’ families, RACHs and their staff, and the regulatory environment regarding psychotropic prescribing |
| Family pressure: if you don't prescribe this medication, the facility is going to send Mum away. |
| *The GPs are faced with a very difficult situation where the families are saying if you don't prescribe this medication the facility is going to send Mum away and not allow her to come back. And I've also experienced it where people have said to me Well, mum can't stay there if you don't say she can have this medication. … But we give her a little bit more medication.* ***(P1, Geriatrician)*** *There are families that are complaining about this particular person's behaviour. You've got to do something and … doctors get caught in that expectation that they will fix this problem and so psychotropic tend to be a quick go to.* ***(P2, Psychiatrist)*** |
| Staff pressure: we can't keep this man because he's hitting our staff or hitting other residents. |
| *“So, there's almost an emotional blackmail happening, with staff saying, well, we can't keep this man because he's hitting our staff or hitting other residents, which is even worse unless you give him something to quiet him down and I would hear that regularly.* ***(P1, Geriatrician)*** *And I think often it's pressure from staff that they say to the doctor, we need these drugs and then someone writes it up, I think that you've got to be pretty reluctant to do it as a doctor. I mean, I think decide that it's needed, or it's not needed.* ***(P3, Geriatrician).*** *They[staff] just … glaze over and what they want you to do is get script pad out of your book, … and write a drug. You've got nursing care staff who just want them to be calm and settled.* ***(P12, Geriatrician)*** |
| **RACH pressure** |
| *Because aggression towards another resident, or aggression to a staff member will often mean that that person will be put in an ambulance and sent to the nearest hospital with a message saying don't send them back then, sometimes, …you might think OK, in order to keep the person in the facility, we will give them a small dose of medication to see if that will settle them …the reason you're putting them on this is because if you don't, they will have to leave their facility and then basically, they're homeless, so that form of emotional blackmail, I call it that is quite strong.* ***(P1, Geriatrician)*** *So, there's oftentimes …great pressure within the aged care system to do something about this (BPSD) problem. This problem is disrupting life in the care home. It's making it difficult for care staff. It's making it difficult for another residents.* ***(P2, Psychiatrist)*** |
| Systemic barrier: regulatory environment. |
| *So that would be a time. And when it was the outside circumstances that dictate why you should use medication, knowing that with the new regulations that are in place through the aged care quality and Safety Commission and the serious Incident Response scheme, which means you have to report any push or shove or any. Even minor aggressive behaviour has to be reported … a lot of facilities are very reluctant to care for someone who may be aggressive towards another person because that then triggers a serious incident response report.* ***(P1, Geriatrician)*** *They (staff) feel more reassured when the person's got more medication because they perceive that these incidents are less likely to happen. … some of the government requirements makes them(staff) fearful.* ***(P3, Geriatrician)*** |
| Non-involvement and blame shifting regarding psychotropic deprescribing |
| Non-involvement and blame-shifting to other doctors |
| *I often hear about cases where the GP hasn't reviewed somebody for several months, but they've been on several medications Once you get to that stage, it's very difficult then for a doctor to start to unpack polypharmacy. They (GPs) have got a potential limitation in their own sort of confidence, knowledge, skill set. (****P2, Psychiatrist).*** *…you'll inherit somebody's on several psychotropics from a hospital. He doesn't give you any information.* ***(P12, Geriatrician)*** *Majority of the …prescribing is happening before they are coming into residential…they will come up with a big list of medications (****P14, GP).*** |
| Blame shifted onto RACHs and their staff, residents and their families, and regulations around incident reporting |
| Organisational culture |
| *Clinical factors might not be addressed. So, the person might remain with untreated pain, for instance. That might be actually contributing to. The behaviours and psychological symptoms, but that might not be treated so, but psychotropics might actually have been prescribed and so they will then be perpetuated because those other factors are not ameliorated. And then There's the cultural factors and the system barriers that exist within the aged care system that perpetuate the use of psychotropics beyond what would be reasonable or beyond what the guidelines might suggest is an appropriate goal.Should we stop this medication? Is this doing what we want to do? Is it doing something harmful? Is there a side effect that we should be concerned about? … those are things that often don't happen in residential aged care because of the cultural and system challenges and limitations (****P2, Psychiatrist)*** *Organisation that runs in nursing homes… have to promote that [deprescribing] in the culture. So, it really depends on the culture of the organisation you're working in. (****P3, Geriatrician)*** *Then the environment and the care approach towards the client …so, the agenda of the aged care. (****P5, Psychiatrist)*** *So, the aged care standards around deprescribing will make a huge difference. (****P6, Palliative medicine specialist)*** *Regular monitoring…  requires someone who's gonna see you regularly and the structural barrier from an aged care perspective, is that there aren't…system is actually not structured to do that really to deprescribe it all sorts of situations in aged care, worse than anywhere else. (****P12, Geriatrician)*** |
| *Reluctance to deprescribing by the RACH staff* |
| *Staff … are very reluctant if they've got a person that's happily sedated and does what they ask but can still eat and drink, a quiet patient is much easier to manage… than a wandering…, rummaging intrusive, noisy, patient who then goes and falls over. So, you can see why staff may be reluctant to look at reducing the medication. (****P1, Geriatrician)*** *If you just write PRN and you don't explain, then often the nurses will give it all the time. They continue to do that, … It's easier to look after someone who's asleep. (****P7, GP)*** *There's a very strong inertia in the healthcare system to let sleeping dogs lie. (****P12, Geriatrician)*** *So, you really have to have buying from the staff. (****P3, Geriatrician)*** *I had in the past nursing staff at facilities reluctant to make this trouble or reducing and occasionally GPs as well. (****P13, Geriatrician)*** |
| Reluctance to deprescribing by the care recipient |
| *So, barriers can be the patient themselves, who doesn't want to stop it. If they're still understanding and they know they'll feel better. I had it this morning with a patient who's in hospital and we were talking about the medication, he said he didn't want to stop the antidepressant because it actually made him feel better and there it's unfortunately it's lowering his sodium so.* ***(P1, Geriatrician)*** |
| Reluctance to deprescribing by the families |
| *So, you've really got to convince the family this is the right thing to do. Most families will be happy unless they've faced the situation where the residential care facility says if he behaves like that again he is out. (****P1, Geriatrician)*** *Sometimes family can be a barrier. Sometimes family don't want deprescribing to happen. (****P5, Psychiatrist)*** *Sometimes family who insist on keeping it [antipsychotics] going, yeah. …the family are insistent. (****P8, Psychiatrist)*** *I guess family concern that if you stop them [psychotropics], things will get worse again and it may be harder to regain control, …there is a little bit of the ethos, if you like that look, it's working, don't drop the phone, let sleeping dogs lie, keeping it… there's always a lot of a degree of therapeutic inertia, I mean... I see families who are very worried about the degree of frustration for their loved ones it's[deprescribing] causing …when you stop the sedating medications, they wake up and they can suddenly start doing these things [not eating or drinking, being fed and getting aspiration]. (****P10, Geriatrician)*** *We've gotta be humble enough to know that we need to involve the patient possible and the family members… if somebody refuses consent to change a form of therapy …you can't really just force … when you can't guarantee that… there won't be an adverse. (****P11, GP)*** *But it's also families I would never attempt a drug deprescribing, unless I've got absolute buying from the family because. The moment they have any doubts that you know, if anything happens, they'll just warming back on the drug again. So, you know, I often point attempts to, unless the family are really asking for it … families have enormous faith in medication, … letting tablets that keep alive when they're not really. (****P12, Geriatrician)*** *From the family relative perspective, it might be an understanding of what the medications doing, so sometimes this is addressing what they think it's doing. (****P13, Geriatrician)*** *Deprescribing … can be challenging when the residents you know are quite stable and the family is very much on board with all the prescription that's happened then they don't want you to …mix or ….mess up with the medications. too much … you just don't rock the boat too much. (****P14, GP)*** |
| Systemic barrier: regulatory environment (E.G., staff worry about blowback from reported incidents) |
| *Yes. So, I think that some of the government's requirements for reporting make staff quite fearful about weaning drugs in case they feel like they're going to get in trouble if something bad happens.* *… if you don't have the support of the staff and they don't feel safe in doing that and they're feeling anxious or fearful that there'll be an incident, I think it's very hard to wean the drugs. (****P3, Geriatrician)*** *Nurses are insistent as well, …they wish you to continue. … they have responded, and they don't have side effects that I will continue, yeah****. (P8, Psychiatrist)*** *When people are stable there, there's a tendency to say, well, don't rock the boat. (****P11, GP****) Also, if there's ongoing symptoms… agitation or aggression… staff are reluctant to withdraw a medication … there might be more likely to remain on a medication. (****P5, Psychiatrist)*** |
| Non-involvement and blame shifting regarding non-pharmacological prescribing |
| *I to be honest, I don't try to. I've given up trying to get major nondrug treatments. … first of all, … I'm not a nurse or enough to it, so it's not. My core practice, I* don't have *the skills of doing complete skills of doing those sorts of behavioural assessments and the aged care sector has no one really in it. (****P12, Geriatrician)***  *They're[doctors] just simply not trained in non-pharmacological interventions. So, it makes sense that …they're not likely to go to those as a first line of prescription because they don't really know what to say or do? (****P2, Psychiatrist)*** *And a lot of physicians feel that really psychotropic medications are the first line.* *(****P1, Geriatrician)*** |
