# Supplementary material for: Psychotropic medications versus non-pharmacologic approaches for managing behavioural and psychological symptoms in Australian aged care residents with dementia: general practitioners’ and physicians’ perspectives
Source: Ther Adv Psychopharmacol. 2025 Oct 28;15:20451253251387908. doi: 10.1177/20451253251387908 (PMC12575986; doi:10.1177/20451253251387908)
Supplement: sj-docx-5-tpp-10.1177_20451253251387908 – Supplemental material for Psychotropic medications versus non-pharmacologic approaches for managing behavioural and psychological symptoms in Australian aged care residents with dementia: general practitioners’ and physicians’ perspectives [file sj-docx-5-tpp-10.1177_20451253251387908.docx]

# Supplementary material 3: Reasons for Psychotropic medication overprescribing

# Supporting Information 3: Reasons for Psychotropic Medication Overprescribing

| Causes of psychotropic overprescribing |
| --- |
| Difficulty in determining when psychotropic medication is necessary. |
| *It's difficult to get the timing of PRN medications correct. Yeah, it's given too early when they don't really need it and you should be persevering with the non-pharmacological approaches. (****P10, Geriatrician)*** |
| Structural barrier: financial issue |
| … *we don't subsidise the CBT [cognitive behavioural therapy].* ***(P12, Geriatrician)*** |
| Structural barrier: lack of infrastructure for NPIs |
| *… there's no infrastructure to actually map that person's behaviour … So, what are you gonna do? You're just gonna keep running the scripts… there is this huge structural barrier to the use of non-pharmacological prescribing as opposed to pharmacological.* *So, there's a lot of structural reasons why we have these sorts of situations.* ***(P12, Geriatrician)*** |
| Structural barriers: lack of data linkage |
| *Because if people are using private scripts, there's no real record other than the residential aged care facility saying no, we have a patient on this. It's interesting.* *Different GPs use the medications differently, certainly in rural areas there is not the access to geriatricians or to psychiatrists. (****P1, Geriatrician)*** |
| Feasibility/convenience of PRN options over NPIs |
| *So, if you've got a PRN psychotropic on your medication chart sometimes care homes will default to using the PRN rather than actually thinking about how they might implement a very good quality non-pharmacological intervention. (****P2, Psychiatrist)*** *When the GP isn't present on site, so sometimes the regular might get stopped but the PRN one will still be charted there. … when there's… no one present and at 2:00 in the morning, it's hard for facilities and the nurses there to get medical attention. And if they've got something there, they could, you know, use it as needed.*  (***P9, Geriatrician)*** *Often medications are tried first or other responses, rather than trying anything. (****P10, Geriatrician)*** *Absolutely. I think that's (use of PRN) a risk with nursing in general, that's sort of beyond even all this every time RN nurses tend to like to go into their routines. …look overnight there's a higher risk of. Nurses just trying to get the patient to sleep, …but I think when you've got that ratio of one nurse to six patients and plus, they are more tempted to make it a little bit easier in that respect. It's not the best practice, but it can happen. (****P15, GP)*** |
