# Supplementary material for: Psychotropic medications versus non-pharmacologic approaches for managing behavioural and psychological symptoms in Australian aged care residents with dementia: general practitioners’ and physicians’ perspectives
Source: Ther Adv Psychopharmacol. 2025 Oct 28;15:20451253251387908. doi: 10.1177/20451253251387908 (PMC12575986; doi:10.1177/20451253251387908)
Supplement: sj-docx-6-tpp-10.1177_20451253251387908 – Supplemental material for Psychotropic medications versus non-pharmacologic approaches for managing behavioural and psychological symptoms in Australian aged care residents with dementia: general practitioners’ and physicians’ perspectives [file sj-docx-6-tpp-10.1177_20451253251387908.docx]

# Supplementary material 4: Barriers to psychotropic deprescribing

# Supporting Information 4: Barriers to psychotropic deprescribing

| Barriers to psychotropic deprescribing |
| --- |
| Lack of resources |
| Systemic barrier: Lack of access to GPs or specialist review |
| *Lack of access to doctors in residential care, … in regional areas, they might have a GP who visits once a month or something, or an agency who does telehealth or there's not a regular GP. …then no one's taking ownership of reducing the drugs. (****P3, Geriatrician)*** *That[deprescribing] can be made easier if you have a medical team looking at someone, let's say a geriatrician or an expert … if it can be judged whether the risk of making a change is going to be overall beneficial for the individual patient. (****P11, GP)*** *There's even less medical input, less pharmacy input or less … expert… there aren't enough specialists to go around to follow everyone with BPSD in a nursing home… to be honest, you have to go out and see them. You have to actually help …As a specialist, or else GP, … they're often not seeing these people regularly. … it's very hard for someone to get traditional primary care … they're not seeing their GPs once a month. They're seeing GP when there's a problem. (****P12, Geriatrician)*** *So if the nursing home knows that if something goes wrong, they can always get help from somewhere, so they can call the emergency department. So all those things are really helpful. So, you know, it's easy to deprescribe then because you know help is available if something goes wrong, you know. So in Metro area, it's easier that way, you know.* *In a rural area … Doctor ...is on call all the time…So I think maybe that's when the deprescribing might be a bit difficult … they[rural nursing home] would choose to just leave it on. (****P14, GP)*** |
| Systemic barrier: Lack of time among GPs |
| *So if the GP starts a medication they should be interested in is this medication. Is there an adverse side effect? So an actual follow up? there should be Constant and continual follow-up is very difficult in residential aged care because GPs won’t … they have got a lot of people to see… haven't got a lot of time. And that is GPs at time poor they have a lot of people to look after. (****P2, Psychiatrist)*** *Deprescribing in general takes time and it does fall largely on to GP…they've got very short time … my GP colleagues would really struggle to have that time. (****P9, Geriatrician)*** *And it's maybe related to time pressures … for doing this sort of work. (****P10, Geriatrician)*** *Even if you go to deprescribe, it's actually as much work, possibly more work than prescribing and we don't really have a primary care system that supports that to do what I think is actually quite an intensive medical work to do it. (****P12, Geriatrician)*** *The barriers for deprescribing is a clinician time. (****P14, GP)*** |
| Structural barrier: insufficient financial incentives (Inadequate remunerations for GPs) |
| *GP… retreated from aged care because of the remuneration and the fact that it impacts on your ability to run your office space practice. (****P11, GP)*** *Look, remuneration is not that great… so I mean in simple words, if I sit in the clinic and see patients face to face and for the same time I spend in the nursing home, I think I can make more money when I'm seeing patients in the clinic. (****P14, GP)*** |
| Structural barrier: Lack of access to continuum of care from specialists |
| Structural barrier: lack of interpersonal continuity of care |
| *My experience is … these agents get started with their specialist involved in the clients’ care, but then the specialist doesn't remain involved and …the primary care practitioners … or the aged care homes not confident in withdrawing an agent …. then people just remain on things.* (**P5, Psychiatrist)** *…they [residents with a big list of psychotropic medications] will already have the antipsychotics charted in there.... So, when they come into nursing home, … we have to catch the tail about … why was it started? … because … the patient not coming introduced himself to me and … I don't have access to all the big past history. (****P14, GP***) |
| Structural barrier: Lack of informational continuity of care: |
| *And we may or may not be. If you explain to someone who would say. Your mother's. And your mother was put on this medication and often it can be very hard to know what the history of the medication is, how long it's been going for, what does have been used in the past because our medical record systems. That do not easily give that data. (****P11, GP)*** *But you just the GP. He's taken on a new resident. He's not gonna, he or she's not gonna have access to that information, they're not gonna know what drug prescribing that it all started. (****P12, Geriatrician)*** “*The multiple drugs for prescribing is that is that journey from home to care as they're developing behavioural problems and we don't have a strategy once they get into care about deescalating it again.* (**P12, Geriatrician)** *…* |
|  |
|  |
| Intention to avoid the negative consequences of deprescribing. |
| Fear of symptom recurrence |
| *Fear on the part of the doctor that once they have started something, that if they stop it, the behaviour will occur. (****P4, Psychiatrist)*** *If the person still wandering around being very aggressive and hitting people, OK, you can't stop that medication. (****P7, GP)*** *You can have someone who's been briefly settled, and then you start weaning, then suddenly start getting agitated again… there's always risks that you can get recurrence of previously managed symptoms. (****P10, Geriatrician)*** *So, we said, why don't we start deprescribing… we said, look, let's cut down the midday into half as well, then just leave half. You know TDS, but surprisingly, there was incidents that started happening with him being aggressive against other residents …In some residents, the symptoms are obviously… happening mostly …every 2 to 3 days …So those times obviously you can’t deprescribe, you leave it on as PRN. (****P14, GP)*** |
| Physicians’ reluctance to follow pharmacist recommendations from RMMR |
| *Oftentimes, like my frustration, I suppose with it is that I’ve encountered some really good advice provided by pharmacists. That is then not translated into practice and that’s the big challenge. (****P2, Psychiatrist)*** |
| **Several reasons were identified regarding why physicians are hesitant to follow pharmacist recommendations.** |
| Physicians’ prior experience with the outcome of implementing pharmacist recommendation. |
| *… someone has withdrawn an agent because it was suggested by the pharmacist that was inappropriate. And then the person’s behaviour is deteriorated so that spot the referral. (****P5, Psychiatrist)*** |
| Violation of RMMR referral procedure |
| *And sometimes people seem to be, I think other GP seem to be confused about where the medication management reviews come from. Like it sort of just appeared. And I don't know whether the pharmacist is being requested by the aged care staff to do it, or if there's some relationship between the pharmacist and the aged care home.  But they seem to get done without the request of the prescriber as well, which can see a little seems a little bit odd to me with how it sort of fits in. (****P5, Psychiatrist)*** |
| Wrong perception of physicians about importance of pharmacists for RMMR |
| *So, the problem is with that it's (RMMR) a very good program. And certainly, when in there, in an area where there's no specialist and the GPs aren't heavily involved, it definitely improves the quality-of-care. (****P3, Geriatrician)*** |
| The misconception among physicians that RMMR is all about remuneration. |
| *But the other thing with that is that program is remunerated by the GP gets 100 bucks (dollar) from doing it and the pharmacist gets 100 bucks and it's transactional. (****P3, Geriatrician)*** |
| Lack of knowledge and experience among prescriber and care staff |
| *Barriers are knowledge and experience of the prescriber to deprescribing. (****P5, Psychiatrist)*** *I find it much more difficult in a setting where the staff have less education about cleaning of drugs. (****P3, Geriatrician)*** *I don't think any of us have ever been really well trained to deprescribe. (****P11, GP)*** *They've [GPs] got a potential limitation in their own sort of confidence, knowledge, skill set that care homes may not bring people back to their attention to be reviewed in an appropriate time frame. (****P2, Psychiatrist)*** *A General practitioner involved. It doesn't have experience with psychiatry and I've seen that. So, my colleagues have told me it would be supervisors at the time, or GP registrars and junior doctors that say I've not done psychiatry. (****P15, GP)*** |
| Lack of flexible solid dosage forms |
| *Zoloft (sertraline) comes in 50s numbers, for example. Perhaps you don't have; you don't have a way of going down 5 milligrams at a time … we don't have the tools that we need to be training on how to deprescribe different classes of drugs and the access to those forms that would enable us… with those forms solid, you're really stuck unless you have a compounding pharmacist to make it up for you. (****P11, GP)*** |
| Lack of guidance to psychotropic medication deprescribing |
| *I don't think there's really good guidance at that point about how to withdraw, what the risks the withdrawal are. We actually don't really know. … We don't have given clear guidance how to manage drug withdrawal? It's not clear to me if there are withdrawal syndromes from stopping antipsychotic in dementia. I'm told in theory there aren't. (****P12, Geriatrician)*** *I think if there are clear guideline, obviously that helps that we can always revert back to the guidelines that look you know this is not something that we will prescribe in the long term. It's only for a short duration. (****P14, GP)*** *But I think there needs to be a clear plan for reducing and eventually withdrawing them [psychotropics]. I think GPs are good at continuing medications that have been started, but they're not always as good at both up and down titrating medications.* ***(P10, Geriatrician)*** |
