# Supplementary material for: Psychotropic medications versus non-pharmacologic approaches for managing behavioural and psychological symptoms in Australian aged care residents with dementia: general practitioners’ and physicians’ perspectives
Source: Ther Adv Psychopharmacol. 2025 Oct 28;15:20451253251387908. doi: 10.1177/20451253251387908 (PMC12575986; doi:10.1177/20451253251387908)
Supplement: sj-docx-7-tpp-10.1177_20451253251387908 – Supplemental material for Psychotropic medications versus non-pharmacologic approaches for managing behavioural and psychological symptoms in Australian aged care residents with dementia: general practitioners’ and physicians’ perspectives [file sj-docx-7-tpp-10.1177_20451253251387908.docx]

# Supplementary material 5: Barriers to prescribing non-pharmacological interventions

# Supporting Information 5: Barriers to prescribing non-pharmacological interventions

| Barriers to prescribing non-pharmacological interventions. |
| --- |
| Lack of competence in NPIs among doctors and RACH staff |
| Knowledge gaps among doctors and RACH staff |
| *Doctors and medical staff not really having the knowledge to actually implement non-pharmacological interventions with any consistency …criticisms equally valid to residential aged care staff as well, I believe and have less knowledge than the doctors about those interventions. (P****4, Psychiatrist)*** *A lot of physicians don't understand that non-pharmacological measures can be effective… there's a number of reasons why you know, the non-pharmacological management doesn't always work … knowledge of staff would be a very big reason. (****P1, Geriatrician)*** *Well, some of the barriers would be lack of knowledge you know they[doctors] just may not have the knowledge. (****P6, Palliative medicine specialist)*** *I think partly it's knowledge and experience. So, when I went through my training as an old age psychiatrist, there wasn't very much emphasis on non-pharmacological interventions, …So, I think it's partly that lack of familiarity in training.* (***P5, Psychiatrist)*** *There's a range of understanding amongst nurses and allied health in residential aged care…about how to do non-pharmacological management so that's one thing. (****P7, GP)*** *They [GPs] don't have the knowledge and they don't have the confidence I guess to do it [NPI]. And nurses aren't very good at doing this either. And so, there's a big gap, yeah. (****P8, Psychiatrist)*** *I guess lack of knowledge [physician]. … Lack of awareness of the evidence base for what does work.* ***(P10, Geriatrician)*** *Lack of awareness of the effectiveness of non-pharmacological measures. (****P13, Geriatrician)*** |
| Lack of expertise among physicians and RACH staff |
| *And they (RACH staff) don't necessarily have the expertise to do things like take the person for a walk instead of sedating them. (****P1, Geriatrician)*** *Lack of skills … by the staff to implement it. … Lack of trust in the staff’s ability to provide it [NPI]. (****P10, Geriatrician)*** *But they're[doctors] not experts in non-pharmacological interventions and they[doctors] don't necessarily know. (****P2, Psychiatrist)*** *GPs don't have the expertise. (****P8, Psychiatrist)*** |
| Lack of training and education |
| *There's very little training getting to dementia, …calm approach reapproach when they say no to personal care… if the staff don't have the training …no one can do it. (****P3, Geriatrician)*** “*A lack of training and education about those (non-pharmacological) interventions during their[doctors] undergraduate medical training and subsequent specialist training. …even their postgraduate specialty training doesn't really equip them with their understanding of A. how to implement non-pharmacological measures and B. how to assess which non-pharmacological measures may or may not be appropriate.* (**P4, Psychiatrist)** |
| Physicians’ trust in the role of medication and distrust in the effectiveness of NPIs |
| Nature of physician training (culture of doctors’ training) |
| *The doctors are trained in a medical model. They are prescribers and a lot of the time; the medications are the tools of their trade. That's what they know. Well, that's what they that's what they have in their doctor's bag. They've got their script pad, or you know, that's the language that they're sort of very familiar with. It's what they've always done*…*But they do know out of you know, write up 50 milligrams of quetiapine or whatever it might be that they've got on the top of their mind. (****P2, Psychiatrist)*** *It's probably the capacity of care home staff to be able to implement non-pharmacological mechanisms as well. …GPs aren't necessarily responsible for the non-pharmacological interventions you know, for example changing the lighting or, you know, painting something or changing the orientation of the bed, or the care approach. (****P5, Psychiatrist)*** *I am a doctor and … by our own biases we always do think to prescribe. (****P12, Geriatrician)*** |
| Unfamiliarity with the limited effectiveness of drugs |
| *So, I think also, you know culturally, there's been a lack of awareness of the that drugs don't work. And I think that's only starting to come in now. (P****3, Geriatrician)*** |
| Physicians’ distrust of the effectiveness of NPIs |
| *I think the evidence for the use of other things such as music therapy or exercise is not so well known. (****P1, Geriatrician)*** *Lack of trust in the intervention. (****P10, Geriatrician)*** *It would be lack of security. I guess a lack of response to non-pharmacological methods is another factor in in itself. So, if you've trialled most things non-pharmacologically and the patient is not improving, there's quite probably you may have to go down a pharmacological route. (****P15, GP).*** |
| Resource constraints |
| Inadequate trained staff |
| *Prescriptions for non-pharmacological rely on more staffing than we have, so someone might need you know several hours of one-on-one time and in residential care that's just not possible given the ratios. I think also the training of staff. (****P3, Geriatrician) A*** *lot will depend on staffing levels to be able to carry out the non-pharmacological treatment for symptoms and that is of course a huge problem today because staffing numbers are low****…****So, it's there's a number of reasons why you know, the non-pharmacological management doesn't always work. And staffing and resources and knowledge of staff would be a very big reason* ***…****The facilities are often reluctant to refer to dementia service because they know what they will suggest that they should do as non-pharmacological measures. And they don't want to do that because they don't have the staff. (****P1, Geriatrician)*** *You have to have enough staff around to also do it as well. … in the ideal world. It (NPI) takes resources and staffing and it takes staff away from other residents. (****P9, Geriatrician)*** *I think things like lack of consistency in staffing does have an impact. (****P10, Geriatrician)*** *It's about the quality of and interest of the staff. How well trained and supported they are, what ancillary staff that aged care facility has in terms of activity coordinators and activities people. (****P11, GP)*** *Sing along… people coming along to play music, but that's not music therapy, you know, music therapy is about a therapist engage an individual. And I think that is vastly underdone. I mean the realities are there aren't a lot of trainers and therapists in Australia. (****P12, Geriatrician)*** |
| Lack of access to expert support. |
| *Lack of access to support to implement it (NPI). Whether that's through, you know, dementia services or, you know, local geriatric services. (****P10, Geriatrician)*** *The only service that does supposedly provided is the [dementia service]. My general experience with that is it's pretty cookie-cutter. It's not terribly individualised and it's all done on the phone, whereas I think the reality is that probably the most of you will actually be in the environment and working with people. …It's you gotta have those non doctor experts and they're actually gonna be quite widely available and easy to. Access so they're highly restricted and you know, really cheap fashion on the phone virtually. Essentially which I don't think it's an equivalent. I know you all, very likely they do fantastic work, but I I'm not Sure, I'm convinced.****(P12, Geriatrician)*** |
| Lack of time |
| *And lack of time, sometimes for it actually takes a lot of time to understand a person well enough to understand what their needs are, what their preferences are, what provides comfort, what's meaningful and it takes time and often you need a dedicated person to take that time. Yeah. So, for prescribers, they very rarely have that time, if you think of a GP, they're not gonna spend hours because it does sometimes take hours doing that, sometimes easier to just prescribe.* (**P6, Palliative medicine specialist)** *It's very time consuming for one thing, so even if doctors could do it, we don't really have the time and it might take half a day or a full day to actually do the assessments to determine which non-pharma measures might be appropriate. So, we're not taught how to do it. Even if we could, we don't have the time to do it. … And we're not an economical way of doing those assessments because medical time is very expensive compared to allied health and nursing time. So, there's structural and training and practical barriers to us doing a better job. (****P4, Psychiatrist)*** *Those interventions are not applied because they demand time and effort and skilled staff to be able to provide …What's really going on? Their assessments are quick and hurried because everything is under a time pressure. From the way that dementia service works, the consultants will go in and they might be nurses or occupational therapists or dementia specialists. And … they will spend hours in the care home observing and watching and thinking about what's going on, understanding what the problems are that are driving the behaviour and doctors don't do that. They simply don't have that opportunity. They don't have that time. And so, they go to the quicker kind of response and they go to what they know as well. (****P2, Psychiatrist)*** *First of all you need someone to do the intervention. We can't do it. As physicians, we're only on the ward for a limited amount of time. So, it needs to be very much at a nursing intervention. and allied health as well …a lot of the interventions take time for the nurses to do them. And there often aren't enough nurses to spend the required time. So, with my lady who was wandering around crying at night, they did a behaviour chart. And if the nurse sat with her for 5 minutes, she would settle. But then, 30 minutes later, she was crying again. And they said we haven't got time to spend 5 minutes with her once or twice an hour. … They didn't have enough staff to do that. (****P7, GP)*** *They (GPs) don't have the time, GP’s will see a patient briefly usually 10 minutes, 15 minutes maximum and then they will leave instructions for the nurses to do something. (****P8, Psychiatrist)*** *So, I guess the biggest thing with non-pharmacological measures is it takes time. Yes, the non-pharmacological measures will be first every time, but in a practical sense, it's not always possible. It Yeah, it takes time. (****P9, Geriatrician)*** *I think the time would be affecting as well. GPs are very busy.* *Nursing staff are very busy. GPs are come in and they go. (****P13, Geriatrician)*** |
| Lack of specialist dementia units |
| *Some facilities, have specialist units like dementia units and others don't and so those that don't have to manage really don't have the option of moving people into higher levels of care given access to the type of environment you know, for example, where your people can go here if they get agitated, they can be allowed to wander around outside because they have a secure garden. (****P11, GP)*** *Really hard and you know the whole structural residential aged care is not designed to provide skilled dementia care. (****P12, Geriatrician)*** *A patient that's in an aged care facility that's in a very busy high stimulation environment. That triggers that patient to become aggressive towards others. Yeah, that's an environmental factor. You know, you have to help look after the patient in the environment in which they're in, whether that's. The number of residents in an area room or the staff mix. (****P13, Geriatrician)*** |
| Lack of funding |
| *There's absolutely a clear role for a non-pharmacological intervention. It's just they're very hard to implement that not subsidised by PBS, we've used other things using rated pets, Dolls and some of those sort of things, diversional therapy type engagements. there are there aren't a lot of trainers and therapists in Australia and it’s also who pays for it…certainly pharmacy and nursing when you consider that and you know from a behavioural management point of view, occupational therapists are really important in this group. So, but there is no funding structure either of the state or Commonwealth level that supports to put that team around the GP or encourage the GP to practice primary care. (****P12, Geriatrician)*** *If there's enough money for everything. Then I think if there's enough money for everything, we need well trained staff who can do the non-pharmacological management. (****P7, GP).*** |
| Nature and severity of behaviours |
| *So probably one would be aggression, the aggressive severity of the patient action is something that you can see quite quickly and it's really you can see escalating quickly to become physical for acts of violence on. Individuals around them, as well as so it would be the degree of aggression…Maybe the nature of delusions or the nature of persecutory content, I think is that it has affected the patient.* *Feeling fearful and that may lead to aggression that may lead to anger bursts. (****P15, GP)*** |
