# Supplementary material for: Psychotropic medications versus non-pharmacologic approaches for managing behavioural and psychological symptoms in Australian aged care residents with dementia: general practitioners’ and physicians’ perspectives
Source: Ther Adv Psychopharmacol. 2025 Oct 28;15:20451253251387908. doi: 10.1177/20451253251387908 (PMC12575986; doi:10.1177/20451253251387908)
Supplement: sj-docx-8-tpp-10.1177_20451253251387908 – Supplemental material for Psychotropic medications versus non-pharmacologic approaches for managing behavioural and psychological symptoms in Australian aged care residents with dementia: general practitioners’ and physicians’ perspectives [file sj-docx-8-tpp-10.1177_20451253251387908.docx]

# Supplementary material 6: Potential solutions suggested to optimise the management of BPSD

# Supporting Information 6: Potential solutions suggested to optimise the management of BPSD

| Potential solutions suggested to optimise the management of BPSD |
| --- |
| **Using incentive strategies** |
| **Increase aged care worker salaries to attract more qualified individuals.** |
| *You still do your six-week PCA [personal care assistant] course, so people don't have the basic learning skills to equip them to take on more advanced levels of training. The way to address that potentially. And we've seen some steps post royal commission is to actually pay aged care workers better so that a higher class of person, preferably with some prevocational training already, or at least a mandated degree of secondary schooling to show an aptitude … gets employed to the sector in the 1st place as an identified career path going forward. (****P4, Psychiatrist)*** |
| Establishing business models for GPs. |
| *... making sure that there is adequate remuneration and that there's a sort of a business model that works for GPs to have enough time to actually or that might not even make them specialists in residential aged care, so my experience is I've worked with a few GP practices who have taken responsibility for being more expert in the care of people with dementia, for that have been more motivated to work within residential aged care and typically again it's sort of anecdotal, but my sense is that that's a model that potentially could enable better practice. Because that's what those doctors do and so, they're thinking about having a skill set that is more aligned with best practice in residential aged care. Whereas you know doctors who add that on to their very busy business and work schedule tend to perhaps not be enabled in the same way, I think that dissemination of clear, simple succinct guidelines is really important. (****P2, Psychiatrist)*** *There's been a change in the way that medical care has been delivered in aged care. There are structural changes going on so such as organisations taking over the medical care whereas GP…somewhat retreated from aged care because of remuneration and the fact that it impacts on your ability to run your office space practice. (****P11, GP)*** |
| **Optimising the workforces** |
| Optimising the workforce of Geriatricians and old age psychiatrists, |
| *Look, I think that increasing the support available through specialists, so actually making it more viable for geriatricians and old age psychiatrists to actually viably and sustainably spend time working within residential aged care contexts, so making you know making sure that the Medicare numbers, for instance, enable private practitioners and potentially you know sort of publicly based practitioners to actually get in and spend time supporting the work of GP. In residential aged care, there's a small workforce of geriatricians and an even smaller workforce of old age psychiatrists, so boosting the capacity of that workforce, I think, would also be an important enabler of improving prescribing practices. (****P2, Psychiatrist)*** *Other approaches look, I guess having access to expertise… to support primary care within residential aged care to be honest, it's in my view is it's, you know… it's the GPs and the staff within the facilities job to manage to reach the day-to-day staff, we're there to provide help and support for you know, intermittent basis for specific problems we can't take on the job of the GP for them you don't have the staff to do. (****P10, Geriatrician)*** |
| Increasing the number of care staff |
| *…most behaviours …can be avoided or managed if you have enough staff, well trained staff that are attuned to the needs of the client, so I guess the barriers to being able to provide that environment are the ones that need to get addressed… that's probably mostly about money and staffing. (****P5, Psychiatrist)*** *But also having extra staffing…dementia service prescribes really good recommendations, but the facility doesn't have the staff to implement what they're suggesting. (****P1, Geriatrician)*** *The places where I work, … the staff are more educated about the lack of long-term efficacy and they're quite motivated to get the drugs reduced. (****P3, Geriatrician)*** *We need well trained staff who can do the non-pharmacological management and who also understand dementia. And we need well trained. Doctors, GP’s and others the same who understand dementia and understand non-pharmacological management. (****P7, GP)*** *And adequate staff ratios within an agent facility… makes a difference to people's experience with the BPSD and their ability to manage symptoms. (****P13, Geriatrician)*** |
| Improving Workforce Knowledge and Skills |
| **Integrating dementia specific knowledge into prevocational qualifications** |
| *What we're doing is chasing our tails a bit as organisations giving training. There we do go into facilities and teach the staff and provide education to GPs as well that you know, I've highlighted the shortcoming of education with the sector where the sector might have at 50% staff turnover in 12 months, … but keep doing it. And unless you entrench the dementia specific knowledge in peoples’ prevocational qualifications, it's a doomed exercise I think around the capacity of the workforce to absorb highly technical and skills-based education as well. (****P4, Psychiatrist)*** |
| Educate and train the aged care and medical staff workforce on NPIs. |
| *Currently you can become a personal care attendant by doing a six-week online course, potentially with one optional module on dementia... that's manifestly inadequate for a group of people who will be required to look after the 70% of residents of aged care who have cognitive impairment and dementia... you can't look after somebody with that problem unless you are taught something about that problem. So, we're not teaching our care staff enough about the measure and non-pharma assessments either made the point about the nurses, who tend to be in charge of these facilities, the PCA's report to the nurses themselves, don't have that training so are unable to guide them. So, the way to address the issues systemically is to educate and train the aged care and the medical staff nursing workforce a lot better before they are let loose on aged care residents at the moment*… *so, Doctors, as I've mentioned, there's virtually no training about dementia, let alone about the BPSD. Nurses, when they do their undergraduate training, they don't get any either. … so, doctors and nurses. And other allied health practitioners need to be better educated on these things.* (***P4, Psychiatrist)*** *I think we need better training for staff. Yeah, around what is dementia, what causes behaviours of dementia? What's the indication for psychotropic drugs and what are the alternatives. (****P3, Geriatrician)*** |
| …*But I think education, teaching residential staff, care staff, that there are other ways there is evidence for music therapy, there is evidence or other, I think that education that medications aren't as effective as people think they are. I think that's always really interesting*…*But I think education is by far the most important thing. Yeah, they've already made it more difficult to prescribe antipsychotics, which is fine, but it's just let everybody to go round the back. Door and simply prescribe on a private script, so clearly just making it harder. … I think. Education is the key to it. (****P1, Geriatrician)*** …*I mean, I think some of the hardest. Hardest situations I see is where Psychotropics have been used in Lewy body dementia with terrible effect, shocking adverse effects and I've seen it once or twice where it's been irreversible and it's so yeah, a little bit education…You would need to establish a kind of readiness for change so sometimes that takes time. Yeah, making sure that people have the sort of skills they need and the opportunities to identify what the strategies are and implementing those strategies and you know the motivation to do it and I think when you start to see the benefits of the non-pharmacological strategies, that's very motivating for staff …In terms of enablers, it would be education. Education for both care workers and nurses so that they don't just reach for medication they try other things. (****P6, Palliative medicine specialist)*** *It has to be education for as I said families, staff. We know that if we. Then. Train staff to do person centred care. Then rates of agitation and aggression drop. If we can involve families in that planning, that's the great. Help as well… it makes economic sense to train the. Is that you know, because behaviours increase the cost of care and if we can manage them better, then it will decrease the cost of care. (****P8, Psychiatrist)*** *Specialised training and ongoing training and ongoing. Support. In how to interact with people with dementia? It's key and critical and you need modelling and leadership around that. … ongoing education about when you should use antipsychotics and when we shouldn't. It's really important.* ***(P13, Geriatrician)*** *We did a study where we successfully deprescribing antipsychotics for 75% of people that were in the trial and we did it by a combination of things. It was nurse education, family education and pharmacist education as well. Yeah. And so that that was helpful. (****P8, Psychiatrist)*** |
| Education as a tool to change attitude of care staff towards psychotropic and NPIs. |
| *So, I suppose it's more education than the, you know, in nursing homes about drugs, whether they work. Non-pharmacological. Approach and also reassurance about some of the government processes that that you know that they're not going to get in trouble if they try to wean something and there's an incident. (****P3, Geriatrician)*** *So, I think it's important for us to let people know there's. A you know. There’re other things you do before reaching for the script pad.* *(****P1, Geriatrician)*** *It comes down to awareness of what is the scientific evidence behind what works. It's around educating staff and actually putting in place proper adult learning based educational programs, I mean. Giving a single talk, giving a hand at setting up a website and an app, it's not actually gonna change behaviour actually put in place proper. You know, behavioural change, educational processes. You know whether that be detailing or or, you know, feedback and audit. It's a little bit more sophisticated than just giving a talk and expecting everyone to change and that's true where it's not part of political or public got to approach. (****P10, Geriatrician)*** |
| Supportive positive education for GPs from specialists |
| *Look, I think that supportive positive education is a potential enabler and I know that there are there, there are various webinars and seminars that people like the. College of GPs offers. Often times, you know, they might reach out to psycho geriatrician to Support some of those education opportunities so and I think that it's really important that education is presented for GP's in a way that is positive, that doesn't sort of tell them off for their prescribing practices, but helps to win them over that. (****P2, Psychiatrist)*** *so supporting doctors who particularly GP's who practice in this area. (****P11, GP)*** |
| Medication repurposing |
| *We had a period where we used morphine liquid so ordine liquid morphine as a behaviour management and that was really, really effective. But then they cut the number of nursing stuff. So , you can't have it in your cupboard cause it's an S8(Schedule 8) and therefore we ended up using. Much more potent medication when actually 2.5 or 3 milligrams of morphine orally, 3 * a day settle people really well. We used to call. It the heroin effect… it was that lovely anti-anxiety effect that morphine can have and I still will prescribe it quite often notin palliative care, but actually as a behavioural. But I can only do it in places where I know there's a registered nurse on for at least. You know, easily available for at least 18 hours. Or 16 hours of the day. So, there's all those little tweaks you can make to medication. (****P1, Geriatrician)*** |
| Government funding for education and staffing |
| *Well, I think it would be good if governments could fund education and staffing of care homes in a better way because I think it's all about the environment and the carer approach to the clients. (****P5, Psychiatrist)*** *If there's enough money for everything, we need well trained staff who can do the non-pharmacological management. (****P7, GP)*** *That the primary care structure so you know the problem is so prevalent that actually it can't just be managed by specialist alone. We need to be intercepted with the functioning primary care sector. And I think the primary care sector for people in residential aged care needs to be multidisciplinary. That needs to bring in the skills of pharmacists, expert nurses. To extent allied health, but certainly pharmacy and nursing when you consider that and you know from a behavioural management point of view, occupational therapists are really important in this group. So but there is no funding structure either of the state or Commonwealth level that supports to put that team around the GP or encourage the GP to practice primary care in a way that. They would, it would be the expectation if they were living in the community, so most older people in the community see their GP regularly. You go to. Residential aged care you. Never see a GP regularly and yet you've got more burden than illness than you ever had before in your life. (****P12, Geriatrician)*** |
| Optimising the nursing homes physical environment |
| *Also, a better design of nursing homes is important and adequate space. And consideration of the mix of residents in the area. So, if you've got residents who. Are always being aggressive with each other. That then perhaps you can separate them and put them in different sections. There are many things like the lighting, the temperature, the access to outdoors. There's, you know, probably a list of 50 things we could do, yeah. (****P8, Psychiatrist)*** *Environmental factors. Overcrowding. Lack of access to quiet space, lack of access to outdoor space. Those issues are not addressed adequately in residential aged care for a variety of reasons. (****P2, Psychiatrist)*** *Given access to the type of environment you know, for example, where your people can go here if they get agitated, they can be allowed to wander around outside because they have a secure garden. (****P11, GP)*** *Environmental factors in the facility is important. Noise and space, adequate opportunities to do stuff. Is important. To help manage the symptoms and focusing on personalised care plans for people and being able to be supported not only to make a plan but to actually enact it, being trained to pick up on those needs that people. … I think this would really help reduce the incidence of symptoms of BPSD and inspire confidence that medications are not always the solution. (****P13, Geriatrician)*** |
| Implementing person centred approach |
| *We need to make person centered care a reality across all nursing homes and not just they pay lip service to it. They say they do it, but they don't actually do it. … So even though they may all say they do it, they may not actually perform it to a good standard. So, there's some of the things that we could do.* *(****P8, Psychiatrist)*** *That we need to learn how to prescribe and deprescribing that each individual person a little bit differently. It's not. It's not a cookbook you put in this much and that much and it's all fixed and then you reduce that much and that much and it's all better. It's not a cookbook. We it's a human being. They've got their own personalities and their own ways of looking at the world, even with dementia and we need to. Take all that into account and the facility has its own needs, its own staffing problems, issues at the moment, everywhere. (****P7, GP)*** |
| Optimising legislative approaches that limit prescribing flexibility. |
| *I do sometimes think that using legislative approaches, particularly for pharmacological agents, is a bit of a sledgehammer to crack a Walnut because you will get it actually limits what your ability to prescribe things which actually do need to be prescribed. And so then you need to have a good balance between ability to use evidence-based medications, but not prescribing things beyond where they should be prescribed. (****P10, Geriatrician)*** *We've seen where patients who’ve very recently come out of hospital. They had severe behavioural status, like quite aggressive, violent, assaulting, you know, staff members and other patients and residents who, because of the label of chemical restraint and the concerns of the aged care provider that. They don't want. That person to be prescribed because if they got reported to the Commonwealth deprescribing abruptly on Arrival and finally they end up back in hospital. Because they're coming listed again. So, I think there's that's the problem is we manage, we don't manage it we the system doesn't manage this in any way really very well. (****P12, Geriatrician)*** |
| Enhancing evidence-based practice approach. |
| *A more, a better and more sophisticated evidence base and I think we need to know what works and what doesn't. I think we need to look at also how you use the implementation science approach to get the education and it and put into practice and sustain the evidence of what works and we gotta stop doing things that don't work. I guess that's probably where I would sit. (****P10, Geriatrician)*** |
| Ensuring continuity of care |
| *And there would be expectation of continuity of care that can or may or may not happen, sort of a range of reasons that's there are these patients moved to a facility outside the geographic range of the doctor. But anyway, there is basically the question of GP based care versus this is sort of some specialists, but doctors, doctors who may or may not be GP's and may or may not have the full breadth of experience of GP's there might be sort of ex junior hospital doctors who have decided that they want to go work for a company that provides paid care services …the knowledge of the patient's long term history and their symptomatology and how they respond, it means that doctors who have that are more likely to be able to make effective predictions of whether this person has a UTI or not, … so the role of continuity of care and that that's a possibly a factor that should be looked at in the context of psychotropic management of behavioural problems in dementia* *…I mean the pharmacists probably tell you better. You know, if you have a single pharmacy for the aged care facility, you know the pharmacist can certainly probably give you a much better idea because they have records of dispensing records of prescribing hundreds of drug shots. So, getting a handle on what has happened in the past, … what's been tried. (****P11, GP)*** *There that ability to make sure that the person earns their antipsychotic medication, I'm not sure is there. And of course, when they transition into care, you usually new GP very little handover and so probably the last thing you're gonna do is change the prescriber, even if it wasn't appropriate. And I think that's this is the dilemma we have that we've got this one-size-fits-all approach to the antipsychotic prescribing. (****P12, Geriatrician)*** |
| Strengthening RMMR |
| *So essentially, you know, it's an excellent service. It's good that it's funded, that it's available. It should be used really it should be used all the time, every resident in, in aged care should know it's available for everybody, but not everybody necessarily, it's referred through. It is a resource that should be optimised and I've seen it be very helpful and it could be much more helpful if there was. Confidence and willingness from doctors to perhaps take on board recommendations from pharmacists and to utilise them. My own personal experience working with pharmacists is also very positive. (****P2, Psychiatrist)*** *I certainly personally bound you very much input from a skilled pharmacist, but I guess there's always a tension between providing good clinical advice versus earning your fee from the government. So, it's I can sort of see that I guess that to take the next step to the new regime… I think it has value. It provides useful extra information that they may well identify things that perhaps doctor as the bottom doesn't realise, hasn't kept up to date with the latest, particularly with your medications.* (***P10, Geriatrician)*** |
| *Look, I think in principle they're a good idea. The dilemma is it's about teamwork and there'll be some GP's that will pass that potential teamwork and they have a good working relationship with the pharmacists that they work with and but actually what would make a difference to prescribing is about the interaction between the doctor and the pharmacist rather than simply doing a pharmacy review, I think having said that, I also think that the dementia support pharmacy review. I think the quality of that can be very variable. I think there can be some that I mean I know some clinical pharmacists who do these things. They do really good review like an excellent pharmacist whose opinion you would trust and you will listen to. And then others and I've seen them.  Like they just they printed off a four-Wheeler and I think that's the problem. There's a there's what's the quality control of the method medication review and you know. But we should not be too cynical, but I'm sure there's some that are done to generate income and there are some that are done to generate the good output. I mean, that's not uncommon in healthcare practice, but at the end of the day, I think how you get the pharmacist to the doctor to interact is actually the key to the success of the medication review. Actually, I think this sort of role in helping to manage deprescribing. I think you get all I would hope you would get 0 pushback from the medical community about that where it's been significant. (****P12, Geriatrician)*** *And I've also seen the results of RMMR's. I think in aged care facilities, I find them really helpful. Yes, that really help. Because our pharmacists will just prompt us to. You know, rethink. Medications that we're using or not using or consider alternatives. Ninety percent of. the time, maybe. We had sorted this out, but it's like 5 to 10% of the time. There will be a new idea that we're looking at this. And that's really great. (****P13, Geriatrician)*** *You know, there are some pharmacists who would do a really good report, which improves your confidence and some pharmacists who are doing the report with a few things here and there. (****P14, GP)*** |
| Collaboration between physicians and other stakeholders |
| *…ideally, we should work together. So, I'll give you another example. Different lady, different facility few years ago. So, this lady. They used to have for an hour on a Thursday a visiting guitar player [young man] would come and play the guitar for everybody and sing…. She had quite advanced dementia, but she was walking around. She liked this young man and she wanted to be in the front. She wanted to be near him. She wanted to talk to him even when he was singing, you know, she was annoying. She was very annoying and the staff said to me, could you please do something so and they meant medication. And so, I sat down with the nurse. The nurse had done some special training in non-pharmacological management. So, we sat down for only maybe 15 minutes. And we talked about this lady. OK, what are we going to do she's like this, she tends to take a shine to people and be very annoying with other people sometimes too. That's her personality. OK. So, we're not going to fix that overnight. She doesn't remember. So, it's no good telling her. Don't do this because she doesn't remember. She doesn't process. OK. Is there anything else she likes to do? So, we talked about different things. She was going for a walk. They don't have enough staff to take her for a walk. She likes to have a cup of tea with a biscuit around about 11:00 in the morning, that's when the guitar player comes. But she likes to have her cup of tea out in the garden. So, what we decided was at 10:45, we take her out to the garden, sit her down with a cup of tea and maybe one or two biscuits. Yeah, she actually couldn't get out of her chair. She actually was too fat, so sat out there. 12:00 He's [guitar player] gone. Bring her back in for lunch. She doesn't know that he's [guitar plyer] been and gone. She hasn't annoyed everyone, you know. So that worked really well. And that's because the nurse and I both understood about just a simple principle of distraction and finding an alternative activity. ….so, we need to just work in the context that we've got with the person and with the facility and work it out. And if we work together, the nurses and the doctors and the pharmacists and the allied health, you know, for some diversional therapy, if we work together, then we, I'm sure we can do this much better. (****P7, GP)*** *We need to have this collaboration with nurses, doctors, pharmacists, as well as the politicians and the bureaucrats. We need to all come together to make sure that, yes, we do work commissions and we're focusing on non-pharmacological, but many patients still have their place in protecting patients and acting in their best. (****P15, GP)****.* |
| Establishing partnerships between doctors and nurses or pharmacists |
| *From a prescribing point of view, there are people who are there seeing them every month and saying, Oh yeah, where's Mr. so and so up to with us like a psychotic. I should cut that back again because she did several the last months and they're so happy. Let's just tweak it down a little lower and gradually reduce it and get rid of it over a few months because that would be the safest way to do it. There will be the reliable way to do it, I suspect. This is sort of, I think from a you know, if we were to create solutions for this, we need to be looking at partnering prescribers with others. So you can have advanced practice in whether it's nurse practitioners, maybe pharmacists who could help do deprescribing and they could you know that would be a way of finding a workforce that could actually do that regular monitoring cause that's actually the real barrier to my mind, is that to do this you actually, if I'm going to do a drug withdrawal or plan withdrawal medications over, see the patient multiple times and it it's a big commitment. … And as I say, it may be that. The solution for the if. You're gonna do this. Is to have. People working in you know whether it's advanced practice pharmacists or nurse practitioners working with prescribers to deprescribing and for that to be. Managed in a joint fashion and. You have that sort of more peripheral oversight. You don't need to physically be doing. It but you could you. Could supervise. (****P12, Geriatrician)*** |
